# Transforming LiTMP Lithiation of Challenging Diazines through Gallium Alkyl Trans‐Metal‐Trapping

**DOI:** 10.1002/anie.201607284

**Published:** 2016-10-06

**Authors:** Marina Uzelac, Alan R. Kennedy, Eva Hevia, Robert E. Mulvey

**Affiliations:** ^1^ WestCHEM, Department of Pure and Applied ChemistryUniversity of StrathclydeGlasgowG1 1XLUK

**Keywords:** diazines, gallium, heterocyclic chemistry, lithiation, structure elucidation

## Abstract

This study establishes a new trans‐metal‐trapping (TMT) procedure based on a mixture of LiTMP (the base) and tris(trimethylsilylmethyl)gallium [Ga(CH_2_SiMe_3_)_3_, GaR_3_] (the trap) that, operating in a tandem manner, is effective for the regioselective deprotonation of sensitive diazines in hydrocarbon solution, as illustrated through reactions of pyrazine, pyridazine, and pyrimidine, as well as through the N‐S heterocycle benzothiazole. The metallo‐activated complexes of all of these compounds were isolated and structurally defined.

As one of the most used strategies in synthetic campaigns, metalation chemistry is currently enjoying a remarkable period of advancement.^1^ Lithium alkyls and amides remain front‐runners for base candidates in routine C−H to C−metal transformations, but for non‐routine, more formidable substrates, base selection is generally far from straightforward.^2^ One of nature's most important class of heterocycles utilized widely in agrochemicals, foodstuffs, pharmaceuticals, and many other commercial commodities, diazines are firmly in the formidable category, especially parent naked diazines devoid of substituents capable of assisting the direction of the metalation.^3^ To avoid competition from nucleophilic addition caused by a low‐lying LUMO in the heteroaromatic ring, bulky LiTMP (TMP is 2,2,6,6‐tetramethylpiperidide) is preferred to more basic alkyllithium reagents as the base for these π‐deficient diazines, as first noted by Quéguiner.^4^ However, lithiated diazine intermediates are generally unstable, so Knochel,^5^ Kondo,^6^ Mongin,^7^ and Hevia^8^ have employed coalitions of components typically but not exclusively based on the softer metal zinc to improve stabilities and to perform metalation under milder conditions. Though excellent progress has been made, these coalition approaches still have their limitations, as for example in the zincation of pyrazine using [(THF)LiZn(TMP)*t*Bu_2_], where no stoichiometric control is possible because only 2,5‐dizincation occurs in a 1:1 base:pyrazine stoichiometry.^8^ Moreover, a lack of definitive structural information still impoverishes understanding of this area, which in the most extreme “black box” cases leads to a misidentification of the actual metalating base.^9^

This paper reports a new trans‐metal‐trapping (TMT) procedure based on a mixture of LiTMP and tris(trimethylsilylmethyl)gallium [Ga(CH_2_SiMe_3_)_3_, GaR_3_] that is effective for regioselective diazine and benzothiazole deprotonation in hydrocarbon solution. Whereas alkyllithium reactions are generally irreversible, LiTMP reactions tend to be p*K*_a_‐dependent equilibria. In TMT, these equilibria are shifted towards the desired lithiated substrate product by its interception by a trapping agent (Scheme 1).^10^

**Scheme 1:**
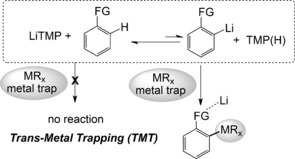
Two‐step lithiation and carbanion trapping process in equilibrium controlling Trans‐Metal‐Trapping (TMT).

We have previously employed the diorganoaluminum amide *i*Bu_2_Al(TMP) as a trapping agent.^10^ For this challenging diazine work, we elected to test GaR_3_ as a trap because its bulkiness compromises its ability to form a weakly basic ate complex with LiTMP and it has the potential to sedate the sensitive incipient carbanions on account of gallium's strong carbophilicity (stronger than that of aluminum). Moreover, this organometallic trap exhibits good hydrocarbon solubility and does not require low temperature procedures, giving it a decided advantage over salt traps (for example, MgCl_2_, ZnCl_2_),^11^ which generally need the use of ethereal solvents and often require low temperatures to avoid competing salt metathesis reactions. This potential has been duly realized through isolation and characterization (crystallographic, elemental analyses, and NMR spectroscopic) of an unprecedented series of gallium diazide and related complexes. As well as launching the concept of gallium TMT, this study reports the first crystal structures of metallodiazine complexes made by metalation (C−H to C−metal) reactions for gallium and indeed, bar one exception for zinc,^8^ for any metal. Furthermore, the study highlights that two‐metal synergistic reactions are not confined to concerted, synchronized processes where the metals belong within the same reagent, but can be extended to tandem, stepwise processes involving two separately added reagents that do not form a co‐complex.

The study first established through NMR spectroscopy that LiTMP and GaR_3_ remain separate in benzene solution^12^ (Supporting Information). Such separation is essential to action the lithiation step of TMT because, whereas free LiTMP is a strong base, combining it with, for example, *i*Bu_3_Al to form aluminate LiAl(TMP)(*i*Bu)_3_ greatly diminishes deprotonating power.10a TMT was then attempted on three classical naked diazines, pyrazine, pyridazine, and pyridimine, as well as the related nitrogen–sulfur ring compound benzothiazole.

Pyrazine previously required four molar equivalents of LiTMP in THF at −75 °C, but only to afford modest yields of 2‐substituted derivatives (39–65 %, depending on the electrophile) mixed in some cases with 2,5‐disubstituted species (16 %).^4^ When performed at room temperature in hexane solution, our new LiTMP‐trialkylgallium approach in a 1:1:1 stoichiometry with pyrazine selectively afforded the 2‐monogallated pyrazine manifested in the crystalline complex [1‐(PMDETA)Li‐3‐(GaR_3_)‐C_4_H_3_N_2_] **1** (isolated yield 61 %: note NMR monitoring of reaction showed **1** is obtained quantitatively, see the Supporting Information).

The role of PMDETA is to aid crystallization and stabilization of the sensitive metallo species by chelating to lithium, but it is added at a later stage to avoid undergoing a competing TMT deprotonation itself. Stoichiometric control was also evident when the base:trap:substrate ratio was doubled to 2:2:1, giving the 2,5‐digallated pyrazine [1,4‐{(PMDETA)Li}_2_‐2,5‐{(GaR_3_)}_2_C_4_H_2_N_2_] **2** cleanly as crystals isolated in a 44 % yield (Scheme 2). However, NMR monitoring of the reaction revealed **2** forms in a 55 % yield, though a second regioisomer is formed in 33 %, which appears to be the analogous product of 2,6‐digallation. This stoichiometric control contrasts with the performance of zincate [(THF)LiZn(TMP)*t*Bu_2_], which operates through a synchronized bimetallic synergy distinct to that of stepwise TMT, as it affords only the 2,5‐disubstituted pyrazine even with a 1:1, base:substrate stoichiometry.^8^ Previously, excess LiTMP (1.5 equivalents) dispensed as 0.5 ZnCl_2_⋅TMEDA/1.5 LiTMP in THF produced 59 % of the isolated 2‐iodopyrazine that reportedly decomposes at room temperature, but in hexane a significant amount of coupled dimer product was also seen.7a Note that a control reaction between pyrazine and gallate LiGaR_4_ 13 did not produce any gallation, but only R group addition, with concomitant dearomatization of the heterocycle (Supporting Information).

**Scheme 2:**
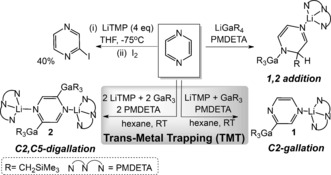
Contrasting reactions of pyrazine with LiTMP and different lithium–gallium combinations.

It may seem surprising that sensitive pyrazinyl mono‐ and di‐carbanions can be trapped in crystalline form at room temperature and structurally defined, but this is where the structures become informative as they show that the heterocyclic units of **1** (Figure 1) and **2** (Figure 2) are cooperatively stabilized through coordination by both the Li and Ga centers that tie up the lone pairs of the N and C atoms, respectively. Both structures are monomeric with aggregation blocked at Li by tridentate PMDETA, though centrosymmetric **2** is tetranuclear having two Ga and two Li centers. Notably, **2** is the more congested structure with its GaR_3_ units having proximal dispositions to the (PMDETA)Li units; whereas in **1** these units have a 1,3‐separation. The Ga−sp^2^C(diazide) bond lengths show little variation with each other or with the Ga−sp^3^C(R group) bonds (see the Supporting Information for full crystallographic details and supporting NMR characterization).

**Figure 1:**
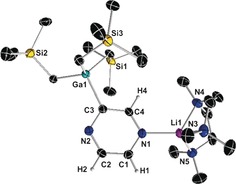
Molecular structure of [1‐(PMDETA)Li‐3‐(GaR_3_)‐C_4_H_3_N_2_] (**1**) with 50 % probability displacement ellipsoids. All H atoms except those in the C_4_H_3_N_2_ ring have been omitted for clarity.

**Figure 2:**
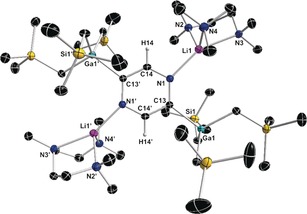
Molecular structure of [1,4‐{(PMDETA)Li}_2_‐2,5‐{(GaR_3_)}_2_C_4_H_2_N_2_] (**2**) with 50 % probability displacement ellipsoids. All H atoms except those in the C_4_H_2_N_2_ ring have been omitted for clarity.

With its 1,2‐placement of N atoms, pyridazine offers a choice of metalation sites. Site selectivity in its metalation is exceptionally challenging, as evident from previous work using excess LiTMP in 0.5 ZnCl_2_⋅TMEDA/1.5 LiTMP/I_2_, which in hexane at room temperature achieved only 27 % of the 3‐iodo product mixed with the 4‐iodo, and 3,6‐diiodo derivatives as well as 54 % unreacted pyridazine (note yields determined from NMR data).7a In THF, the yield of the 3‐iodo product rises to 66 % but only under extreme reflux conditions. On its own, LiTMP (4 equivalents) in THF at −75 °C produced only 16–32 % yields of 3‐substituted pyridazines following quenching with different electrophiles.^4^ When run in hexane solution at room temperature, our TMT reaction afforded a 51 % yield of the isolated product [2‐(PMDETA)Li‐3‐(GaR_3_)‐C_4_H_3_N_2_] **3**. Interestingly, ^1^H NMR monitoring of the reaction revealed an important effect of the order of metal reagent addition on reaction regioselectivity. Thus, when LiTMP is added as a solid to the hexane solution of GaR_3_ and pyridazine, **3** is obtained in a 78 % yield, along with small amounts of the C4‐gallated regioisomer (16 % yield). Contrastingly, if the substrate is added to the hexane suspension of LiTMP and GaR_3_, the yield of **3** decreases to 50 %, and more C4 metalated product is seen (36 %). These contrasting results suggest an activating effect of the GaR_3_ component, which perhaps can initially coordinate to the Lewis basic N atoms of the heterocycle, facilitating its lithiation at C3. The crystal structure of **3** (Figure 3) shows GaR_3_ elects to sit at the most acidic 3‐position adjacent to one N (confirmed in solution by NMR spectra; Supporting Information). A novel feature is the Li(PMDETA) unit bridging the two diazine N atoms [Li−N bond lengths 2.093(5) and 2.043(5) Å for the non‐disordered molecule of the Z′=2 structure] leading to a 1, “2.5”‐separation. Consequently, the spiro Li center, connecting the 3‐ and 2×5‐atom rings, has a 5‐coordinate geometry.

**Figure 3:**
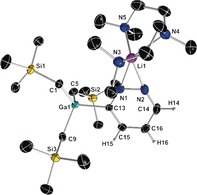
Molecular structure of [2‐(PMDETA)Li‐3‐(GaR_3_)‐C_4_H_3_N_2_] (**3**) with 50 % probability displacement ellipsoids. All H atoms except those in the C_4_H_3_N_2_ ring have been omitted for clarity.

Because pyrimidine was found to be totally inert to LiTMP from 0 °C to reflux temperatures,^4^ it seemed the greatest challenge to TMT. However, as in the case of **1**–**3**, TMT was demonstrated tangibly through isolation and crystallographic characterization of a metalated derivative, here [1‐(PMDETA)Li‐6‐(GaR_3_)‐(C_4_H_3_N_2_)], **4**. This was made in a 27 % isolated crystalline yield, though NMR monitoring of the reaction shows that under the conditions studied **4** forms in a higher 59 % yield. Its structure (Figure S13) exhibits many of the features in **1**–**3** with the proximal 1,6‐separation of its GaR_3_ and (PMDETA)Li akin to that of those in dimetalated pyrazine **2**.

In view of the fact that the 2‐lithiated derivative of the related nitrogen–sulfur heterocycle benzothiazole is known to exist simultaneously in ring‐closed and ring‐open forms, as best evidenced by Boche's ^13^C NMR studies in D_8_‐THF at −75 °C, we extended the TMT study to this fused heterocycle.^14^ Run at room temperature in hexane solution (Scheme 3), an equimolar TMT reaction produced the crystalline complex [2‐(GaR_3_)‐3‐{Li(PMDETA)}C_6_H_4_NCS] **5** (Scheme 3) in a remarkably high isolated yield of 84 %. Significantly, **5** is quantitative in solution with no ring‐opened metallo(2‐isocyano)thiophenolate isomer detected. Deprotonative gallation of the C2 center was evident from its downfield resonance at 209.5 ppm in the ^13^C NMR spectrum. In the crystal, **5** follows the pattern in the TMT diazine series with the GaR_3_ and Li(PMDETA) units adjacent on deprotonated C and N atoms, respectively, with a Ga1‐C13‐N1‐Li1 torsion angle of −13.9(4)° (Figure 4). This first Ga TMT reaction of a N‐S heterocycle is competitive with Mongin's LiTMP/CdCl_2_⋅TMEDA in THF solution approach, which used excess (1.5) base equivalents for a 97 % yield of 2‐iodobenzothiazole after I_2_ quenching, though no metallo intermediate was identified.7b, 15

**Figure 4:**
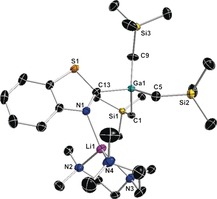
Molecular structure of [2‐(GaR_3_)‐3‐{Li(PMDETA)}C_6_H_4_NCS] (**5**) with 50 % probability displacement ellipsoids. All H atoms have been omitted for clarity.

**Scheme 3:**
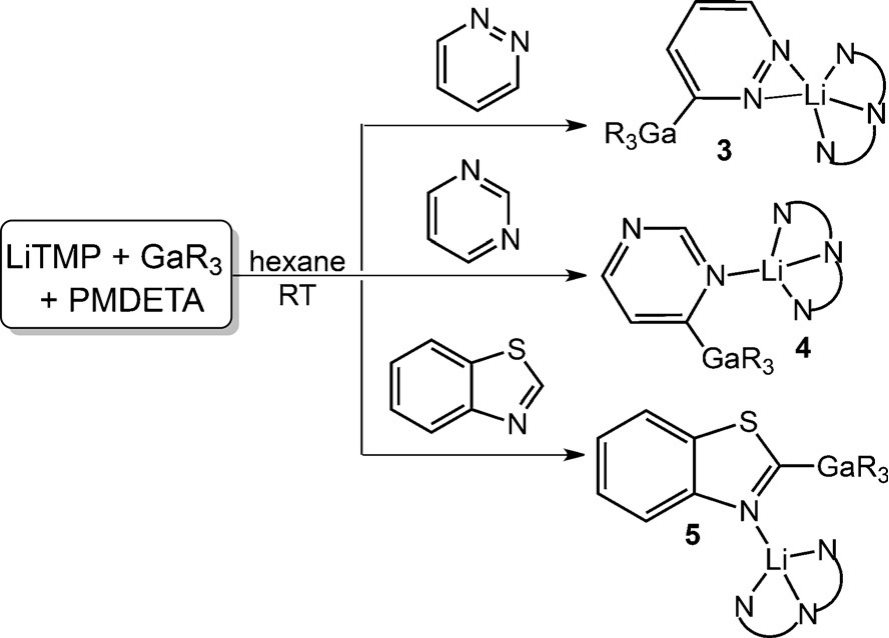
Main products of reactions of TMT mixture with pyridazine, pyrimidine, and benzothiazole.

For synthetic campaigns, it is important that the new C−Ga bonds in these systems are accessible to electrophiles. In preliminary NMR spectroscopic experiments (see the Supporting Information for full details), on subjecting the gallated benzothiazole **5** to methyl trifluoromethanesulfonate (methyl triflate, MeOTf) we obtained a yield of 62 % of the desired methylated product and did not observe any opening of the azole ring. In contrast, though this strong electrophile did quench the monogallated pyrazine **1**, it proved too aggressive and decomposition ensued. Therefore, we turned to a second electrophile in trimethylsilyl chloride, which offers another potential pitfall in being too bulky. Reassuringly, this pitfall proved unfounded and the heterocyclic rings of both **1** and **5** were successfully converted to Me_3_Si derivatives at their C−Ga sites (59 and 88 % yields, respectively). These promising results demand that a full systematic, optimized study of all of these new gallated compounds is now undertaken with a series of electrophiles.

In summary, because LiTMP trans‐metal‐trapping via a gallium alkyl has been substantiated here with challenging sensitive unactivated diazines and benzothiazoles, this can potentially open the floodgates to a general improvement in many other metallation reactions with various sensitive and non‐sensitive substrates where LiTMP and related bulky bases give only low‐to‐moderate yields of products.

## Experimental Section

Full experimental details and copies of NMR spectra are included in the Supporting Information. CCDC 1494979 (**1**), 1494980 (**2**), 1494981 (**3**), 1494982 (**4**), and 1494983 (**5**) contain the supplementary crystallographic data for this paper. These data can be obtained free of charge from The Cambridge Crystallographic Data Centre.

## Supporting information

As a service to our authors and readers, this journal provides supporting information supplied by the authors. Such materials are peer reviewed and may be re‐organized for online delivery, but are not copy‐edited or typeset. Technical support issues arising from supporting information (other than missing files) should be addressed to the authors.

Supplementary
