# Supplementary material for: Transforming LiTMP Lithiation of Challenging Diazines through Gallium Alkyl Trans‐Metal‐Trapping
Source: Angew Chem Int Ed Engl. 2016 Oct 6;55(42):13147–50. doi: 10.1002/anie.201607284 (PMC5113679; doi:10.1002/anie.201607284)
Supplement: Supplementary file 1 — Supplementary [file ANIE-55-13147-s001.pdf]

Angewandte Chemie
Eine Zeitschrift der Gesellschaft Deutscher Chemiker

Supporting Information

Transforming LiTMP Lithiation of Challenging Diazines through Gallium Alkyl Trans-Metal-Trapping

Marina Uzelac, Alan R. Kennedy, Eva Hevia, and Robert E. Mulvey**

anie_201607284_sm_miscellaneous_information.pdf

Contents

Experimental details 1

Synthesis of products 3

GaR_3 and LiTMP mixture 6

NMR spectra of crude mixtures to determine the yields 6

Solid state structures 15

NMR spectra of products 18

Preliminary electrophilic quenching studies 26

Experimental details

General

All reactions were carried out using standard Schlenk and glove box techniques under an inert atmosphere of argon. Solvents (hexane, benzene and toluene) were dried by heating to reflux over sodium benzophenone ketyl and distilled under nitrogen prior to use. *N,N,N',N'',N''*-pentamethyldiethylenetriamine (PMDETA) was dried by heating to reflux over calcium hydride, distilled under nitrogen and stored over 4 Å molecular sieves. TMPH (*N,N,N',N'*-tetramethylpiperidine) and benzothiazole were purchased from Acros Organics and Alfa Aesar, respectively, and stored over 4 Å molecular sieves prior to use. Pyrazine, pyridazine and pyrimidine were purchased from Sigma Aldrich Chemicals, stored in the

glove box and used as received. [$Ga(CH_2SiMe_3)_3$][1] and LiTMP[2] were prepared according to literature methods. NMR spectra were recorded on a Bruker DPX 400 MHz spectrometer, operating at 400.13 MHz for ^1H, and 100.62 MHz for $^{13}C\{^1H\}$. Elemental analyses were obtained using a Perkin Elmer 2400 elemental analyser.

X-Ray Crystallography

Crystallographic data were measured at low temperature using Oxford Diffraction Xcalibur E or Gemini S instruments with graphite-monochromated Mo (λ=0.71073 Å) or Cu (λ=1.54180 Å) radiation. All structures were refined to convergence on F^2 using all unique reflections and programs from the SHELX family.[3] Disorder in one ligand meant that the final model for structure **3** included constraints and restraints imposed on displacement parameters and N-C and C-C distances of the PMDETA ligand of one crystallographically independent molecule. Selected crystallographic data are presented in Table S1 and S2 and full details in cif format can be obtained free of charge from the Cambridge Crystallographic Data Centre via www.ccdc.cam.uk/data_request/cif.

Table S1: Selected crystallographic and refinement parameters for compounds **1-3**.

**compound**	**1**	**2**	**3**
Empirical formula	$C_{25}H_{59}GaLiN_5Si_3$	$C_{46}H_{114}Ga_2Li_2N_8Si_6$	$C_{25}H_{59}GaLiN_5Si_3$
Formula weight	590.70	1101.31	590.70
Crystal system	Monoclinic	Triclinic	Monoclinic
Space group	P 2_1/n	P -1	P 2_1/n
χ (Å)	1.54184	0.71073	0.71073
a (Å)	11.3959(2)	10.8876(7)	25.2264(6)
b (Å)	20.1888(3)	11.6039(9)	10.6825(3)
c (Å)	15.5478(2)	13.7770(6)	27.0136(6)
α (°)	90	101.876(5)	90
β (°)	90.9140(10)	95.764(5)	96.397(2)
γ (°)	90	104.082(6)	90
V ($Å^3$)	3576.62(9)	1631.22(19)	7234.3(3)
Z	4	1	8
Temperature K	123(2)	133(2)	127(2)
μ (mm^{-1})	2.154	0.970	0.880
2θmax (°)	146.53	60.40	60.40
Measured reflections	14000	24932	40054
Unique reflections	6997	8784	18925
Observed reflections	5634	6846	12323
R_{int}	0.0271	0.0477	0.0403
R [on F, obs refln only]	0.0407	0.0357	0.0489
wR [on F^2, all data]	0.1055	0.0724	0.1043
GoF	1.035	0.974	1.009
Largest diff peak/hole (e $Å^{-3}$)	0.691/-0.309	0.573/-0.415	0.696/-0.391

[1] L. M. Dennis, W. Patnode, *J. Am. Chem. Soc.* **1932**, 54, 182.
[2] E. Hevia, A. R. Kennedy, R. E. Mulvey, D. L. Ramsay, S. D. Robertson, *Chem. Eur. J.* **2013**, 19, 14069.
[3] G. M. Sheldrick, Acta Crystallogr., **2008**, *A64*, 112.

Table S2: Selected crystallographic and refinement parameters for compounds **4** and **5**.

**compound**	**4**	**5**
Empirical formula	$C_{25}H_{59}GaLiN_5Si_3$	$C_{28}H_{60}GaLiN_4SSi_3$
Formula weight	590.70	645.79
Crystal system	Monoclinic	Orthorombic
Space group	P 2_1/c	P ca2_1
χ (Å)	0.71073	0.71073
a (Å)	15.9540(12)	18.5734(3)
b (Å)	9.4142(5)	11.9719(2)
c (Å)	24.3742(19)	16.7154(3)
α (°)	90	90
β (°)	98.454(7)	90
γ (°)	90	90
V (Å^3)	3621.1(4)	3716.82(11)
Z	4	4
Temperature K	150(2)	130(2)
μ (mm^{-1})	0.879	0.915
2θmax (°)	56.00	59.98
Measured reflections	18275	54365
Unique reflections	8721	10324
Observed reflections	5314	9394
R_{int}	0.0640	0.0351
R [on F, obs refln only]	0.0639	0.0286
wR [on F^2, all data]	0.1177	0.0677
GoF	1.009	1.044
Largest diff peak/hole (e Å^{-3})	0.534/-0.412	0.782/-0.307

Synthesis of products

1. Synthesis of [1-(PMDETA)Li-3-(GaR_3)-$C_4H_3N_2$] (**1**)

To a suspension of LiTMP (0.074g, 0.5 mmol) and $Ga(CH_2SiMe_3)_3$ (0.165 g, 0.5 mmol) in hexane (10 mL), 1 equivalent of pyrazine (0.04 g, 0.5 mmol) was added *via* solid addition tube at room temperature. As soon as pyrazine was added, a yellow solution was formed which quickly evolved into orange and then red suspension. After stirring for 30 min at room temperature, PMDETA was added (0.11 mL, 0.5 mmol) which induced even stronger precipitation. Addition of 2 mL of toluene and gentle heating afforded solution which upon slow cooling deposited X-ray suitable crystals (0.18 g, 61%). Anal. Calcd for $C_{25}H_{59}GaLiN_5Si_3$: C, 50.83; H, 10.07; N, 11.86. Found: C, 50.05; H, 9.74; N, 11.87.

^{1}H NMR (298 K, *d*$_8$-THF) δ(ppm) -0.82 (6H, s, CH_2SiMe$_3$), -0.18 (27H, s, Si(CH$_3$)$_3$), 2.20 (12H, s, N(CH_3)$_2$), 2.30 (3H, s, NCH_3), 2.39 (4H, br s, NCH_2CH_2N), 2.49 (4H, mult, NCH_2CH_2N), 7.76 (1H, s, pyrazine), 8.48 (1H, s, pyrazine), 8.56 (1H, s, pyrazine). **^{13}C{^{1}H} NMR (298 K, *d*$_8$-THF)** -0.3 (*C*H$_2$SiMe$_3$), 3.5 (Si(*C*H$_3$)$_3$), 43.7, 45.9, 56.1, 58.4 PMDETA,

137.5 (*C*H-pyrazine), 146.4(*C*H-pyrazine), 150.0 (*C*H-pyrazine), 198.8 (*C*-Ga). **^{7}Li NMR (298 K, d_8-THF, reference LiCl in D_2O at 0.00 ppm):** δ 2.35.

2. Synthesis of [1,4-{(PMDETA)Li}$_2$-2,5-{(GaR$_3$)}$_2$C$_4$H$_2$N$_2$] (**2**)

To a suspension of LiTMP (0.074 g, 0.5 mmol) and Ga(CH$_2$SiMe$_3$)$_3$ (0.165 g, 0.5 mmol) in hexane (10 mL), 0.5 equivalent of pyrazine (0.02 g, 0.25 mmol) was added *via* solid addition tube at room temperature. As soon as pyrazine was added, a yellow solution was formed which quickly evolved into orange and then red suspension and finally a green solution. After stirring for 30 min at room temperature, PMDETA was added (0.11 mL, 0.5 mmol) which induced precipitation and a change of colour to orange. Addition of 2 mL of toluene and gentle heating afforded solution which upon slow cooling deposited X-ray suitable crystals (0.12 g, 43.6 %). Anal. Calcd for $C_{46}H_{114}Ga_2Li_2N_8Si_6$: C, 50.17; H, 10.43; N, 10.17. Found: C, 50.47; H, 10.44; N, 9.98.

^{1}H NMR (298 K, d_8-THF) δ(ppm) -0.91 (12H, s, C*H$_2$*SiMe$_3$), -0.15 (54H, s, Si(CH$_3$)$_3$), 2.17 (24H, s, PMDETA-C*H$_3$*), 2.26 (6H, s, PMDETA-C*H$_3$*), 2.34 (8H, mult, PMDETA-C*H$_2$*), 2.45 (8H, mult, PMDETA-C*H$_2$*), 8.58 (2H, s, *H*-pyrazine). **^{13}C{^{1}H} NMR (298 K, d_8-THF)** -0.3 (*C*H$_2$SiMe$_3$), 3.7 (Si(*C*H$_3$)$_3$), 43.8, 46.2, 56.9, 58.7 PMDETA, 153.48 (*C*H-pyrazine), 184.9 (*C*-Ga). **^{7}Li NMR (298 K, d_8-THF, reference LiCl in D_2O at 0.00 ppm)**: δ 2.47.

3. Synthesis of [2-(PMDETA)Li-3-(GaR$_3$)-C$_4$H$_3$N$_2$] (**3**)

To a hexane solution (10 mL) of Ga(CH$_2$SiMe$_3$)$_3$ (0.165 g, 0.5 mmol) and pyridazine (0.04 g, 0.5 mmol), LiTMP (0.074 g, 0.5 mmol) was added *via* solid addition tube at room temperature. As soon as LiTMP was added, a yellow suspension was formed which evolved into orange and then red solution. After stirring for 15 min at room temperature, PMDETA was added (0.11 mL, 0.5 mmol) which induced instant, but short-lived precipitation. Dark red solution was placed at -33 °C to obtain X-ray suitable crystals (0.15 g, 51%). Anal. Calcd for $C_{25}H_{59}GaLiN_5Si_3$: C, 50.83; H, 10.07; N, 11.86. Found: C, 50.34; H, 9.67; N, 12.00.

^{1}H NMR (298 K, d$_8$-THF) δ(ppm) -0.75 (6H, s, CH$_2$SiMe$_3$), -0.16 (27H, s, Si(CH3)$_3$), 2.17 (12H, s, N(CH$_3$)$_2$), 2.41 (7H, mult, NCH$_3$ + NCH$_2$CH$_2$N), 2.54 (4H, mult, NCH$_2$CH$_2$N), 7.17 (1H, s, pyridazine), 7.84 (1H, s, pyridazine), 8.67 (1H, s, pyridazine). **^{13}C{^{1}H} NMR (298 K, d_8-THF)** -0.3 (*C*H$_2$SiMe$_3$), 3.6 (Si(*C*H$_3$)$_3$), 44.1, 45.9, 55.5, 58.1 PMDETA, 122.9 (*C*H-pyridazine), 136.7 (*C*H-pyridazine), 147.4 (*C*H-pyridazine), 199.9 (*C*-Ga). **^{7}Li NMR (298 K, d_8-THF, reference LiCl in D_2O at 0.00 ppm)**: δ 2.80.

4. Synthesis of [1-(PMDETA)Li-6-(GaR_3)-($C_4H_3N_2$)] (**4**)

A hexane solution of pyrimidine (0.04 g, 0.5 mmol in 10 mL hexane) was added *via* syringe at room temperature to a suspension of LiTMP (0.074 g, 0.5 mmol) and $Ga(CH_2SiMe_3)_3$ (0.165 g, 0.5 mmol) in hexane (10 mL). As soon as pyrimidine was added, yellow suspension was formed which evolved into orange and then brown suspension. After stirring for 15 min at room temperature, PMDETA was added (0.11 mL, 0.5 mmol) which induced instant, but short-lived precipitation. The suspension was filtered with cannula and a dark red solution was placed at -33 °C to obtain X-ray suitable crystals overnight (0.08 g, 27%). Anal. Calcd for $C_{25}H_{59}GaLiN_5Si_3$: C, 50.83; H, 10.07; N, 11.86. Found: C, 50.91; H, 10.02; N, 11.82.

^{1}H NMR (298 K, d_8-THF) δ(ppm) -0.83 (6H, s, CH_2SiMe_3), -0.16 (27H, s, $Si(CH3)_3$), 2.20 (12H, s, $N(CH_3)_2$), 2.29 (3H, mult, $NC\mathit{H}_3$), 2.38 (4H, mult, NCH_2CH_2N), 2.49 (4H, mult, NCH_2CH_2N), 7.67 (1H, d, pyrimidine), 7.92 (1H, d, pyrimidine), 8.87 (1H, s, pyrimidine). **^{13}C{^{1}H} NMR (298 K, *d_8*-THF)** -0.4 ($\mathit{C}H_2SiMe_3$), 3.6 ($Si(\mathit{C}H_3)_3$), 43.7, 46.1, 56.4, 55.8 PMDETA, 131.2 (*C*H-pyrimidine), 148.4 (*C*H-pyrimidine), 155.4 (*C*H-pyrimidine), 219.3 (*C*-Ga). **^{7}Li NMR (298 K, *d_8*-THF, reference LiCl in D_2O at 0.00 ppm)**: δ 2.41.

5. Synthesis of [2-(GaR_3)-3-{Li(PMDETA)}C_6H_4NCS] (**5**)

To a suspension of LiTMP (0.074 g, 0.5 mmol) and $Ga(CH_2SiMe_3)_3$ (0.165 g, 0.5 mmol) in hexane (10 mL), 1 equivalent of benzothiazole (0.067 g, 0.5 mmol, 55 μL) was added at room temperature. As soon as benzothiazole was added, a yellow solution was formed which slowly evolved into orange solution. After stirring for 1 hour at room temperature, PMDETA was added (0.11 mL, 0.5 mmol) which induced precipitation. Vigorous heating of the mixture afforded solution which upon slow cooling deposited X-ray suitable crystals (0.27 g, 83.6 %). Anal. Calcd for $C_{28}H_{60}GaLiN_4SSi_3$: C, 52.08; H, 9.37; N, 8.68. Found: C, 52.28; H, 9.15; N, 8.60.

^{1}H NMR (298 K, d_8-THF) δ(ppm) -0.73 (6H, s, $C\mathit{H}_2SiMe_3$), -0.10 (27H, s, $Si(CH_3)_3$), 2.17 (12H, s, PMDETA-$C\mathit{H}_3$), 2.24 (3H, s, PMDETA-$C\mathit{H}_3$), 2.34 (4H, mult, PMDETA-$C\mathit{H}_2$), 2.44 (4H, mult, PMDETA-$C\mathit{H}_2$), 7.02 (1H, t, C*H*-btz), 7.13 (1H, t, C*H*-btz), 7.78 (2H, mult, C*H*-btz). **^{13}C{^{1}H} NMR (298 K, d_8-THF)** 0.9 ($\mathit{C}H_2SiMe_3$), 3.5 ($Si(\mathit{C}H_3)_3$), 43.6 (PMDETA-$\mathit{C}H_3$), 46.0 (PMDETA-CH_3), 56.2(PMDETA-$\mathit{C}H_2$), 58.5 (PMDETA-$\mathit{C}H_2$), 121.4 (*C*H-btz), 121.6 (*C*H-btz), 121.9 (*C*H-btz), 123.3 (*C*H-btz), 139.2 (*C*-btz), 158.6 (*C*-btz), 209.5 (*C*-Ga). **^{7}Li NMR (298 K, *d_8*-THF, reference LiCl in D_2O at 0.00 ppm):** δ 1.87.

GaR_3 and LiTMP mixture

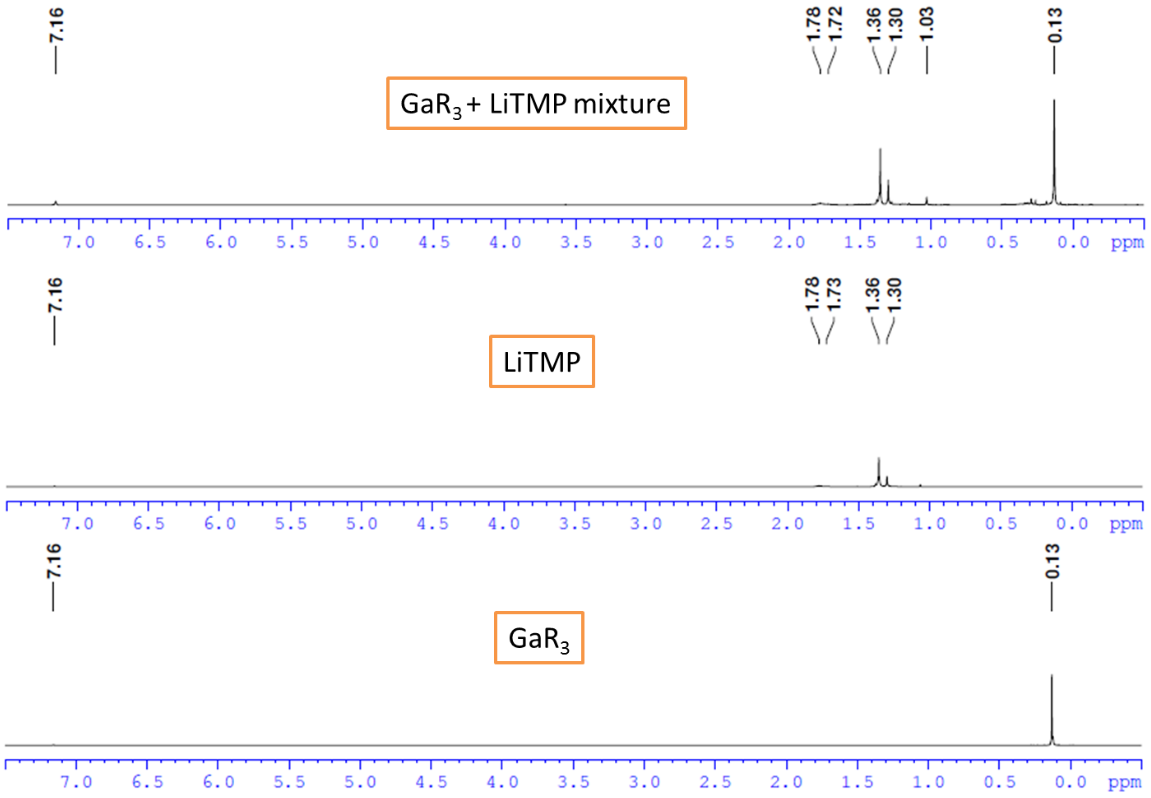

Figure S1: Comparison of ^{1}H NMR spectra in C_6D_6 of pure GaR_3 (bottom), pure LiTMP (middle) and a mixture of GaR_3 and LiTMP (top) revealing no interaction between the two [R = CH_2SiMe_3].

NMR spectra of crude mixtures to determine the yields

1. Monometallation of pyrazine

The reaction was repeated exactly as described in preparation for compound **1** followed by the complete removal of volatiles *in vacuo*. The residue was placed in glovebox, 9 mg of ferrocene was added as an internal standard and the mixture was dissolved in 1 mL of d_8-THF and sealed in Young's tap NMR tube.

The integration *versus* ferrocene revealed 98% of compound **1**, 4% of pyrazine (hydrolysis) and 8% excess of GaR_3 reagent.

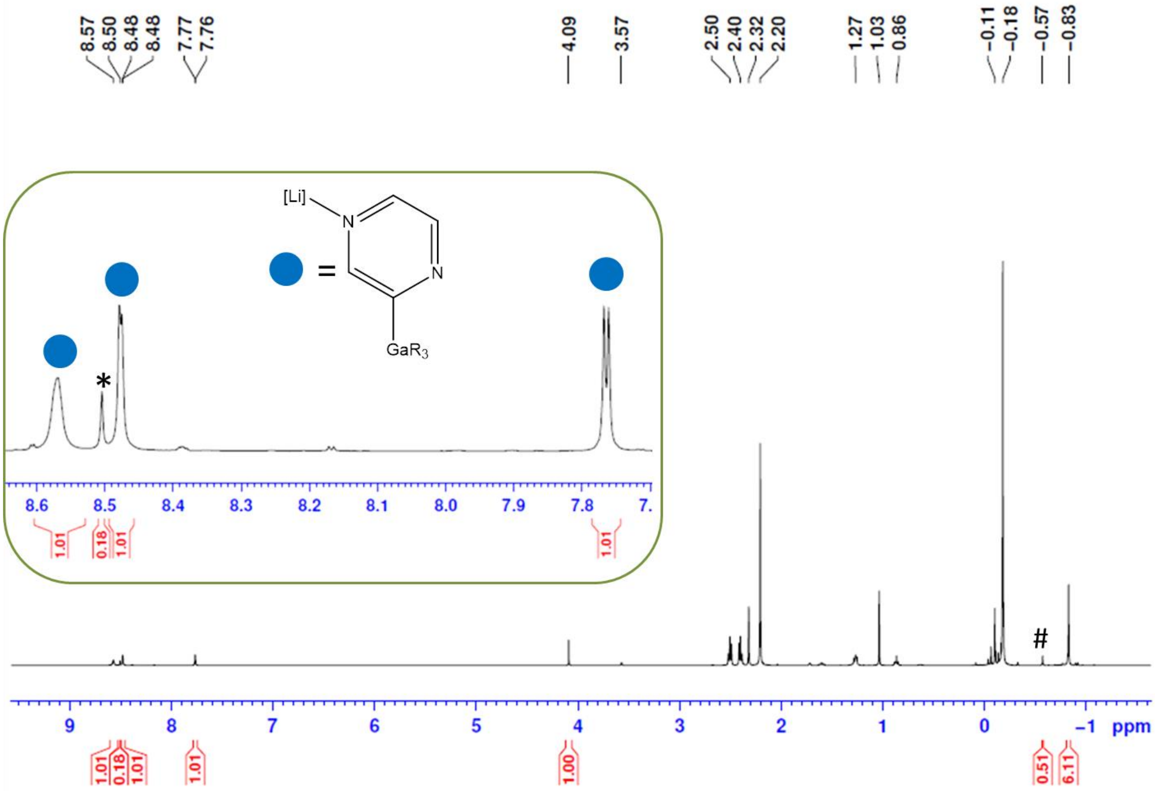

Figure S2: ^{1}H NMR of **1** (98% yield) in d_8-THF solution.

2. Dimetallation of pyrazine

The reaction was repeated exactly as described in preparation for compound **2** followed by the complete removal of volatiles *in vacuo*. The residue was placed in glovebox, 10 mg of ferrocene was added as an internal standard and the mixture was dissolved in 1 mL of d_8-THF and sealed in Young's tap NMR tube.

The integration *versus* ferrocene revealed 55% of compound **2** and 33% of the second regioisomer (2,6-digallated pyrazine).

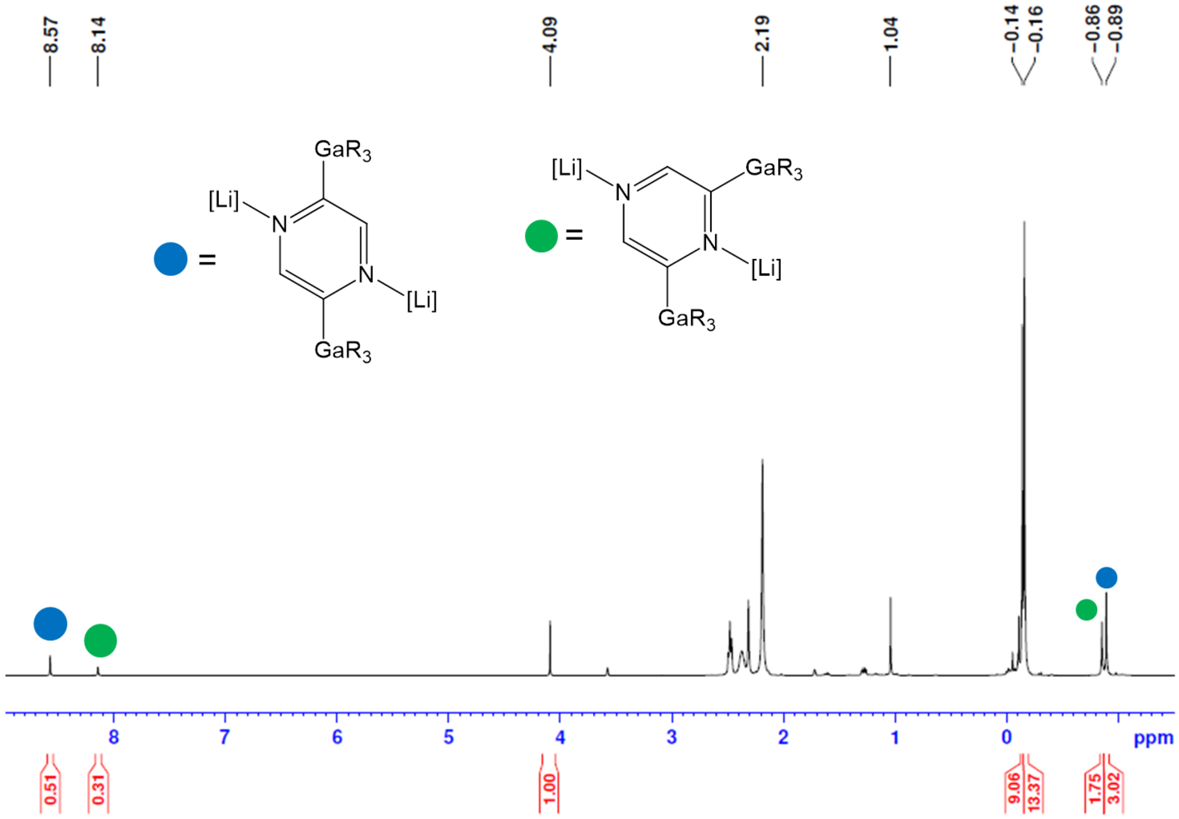

Figure S3: ^{1}H NMR of 2 (55%) in d_8-THF solution.

3. Metallation of pyridazine

   a. The reaction was repeated exactly as described in preparation for compound **3** followed by the complete removal of volatiles *in vacuo*. The residue was placed in glovebox, 9 mg of ferrocene was added as an internal standard and the mixture was dissolved in 1 mL of d8-THF and sealed in Young's tap NMR tube.

      The integration *versus* ferrocene revealed 78% of compound **3**, 16% of the second regioisomer (C4-gallated pyridazine), 52% excess of pyridazine and 21% excess of GaR_3 reagent.

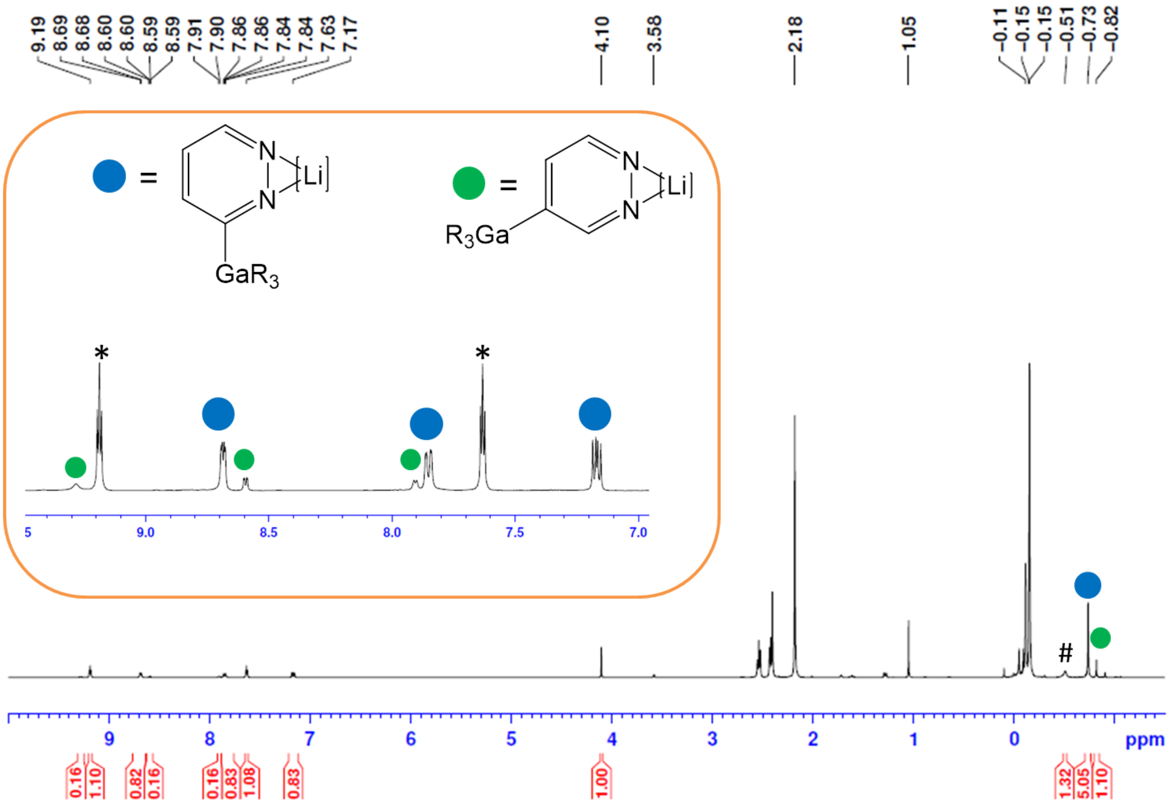

Figure S4: ^{1}H NMR of 3 (78%) in d_8-THF solution.

b. The reaction was performed with a variation in order of addition of reagents. To a suspension of LiTMP (0.07g, 0.5 mmol) and $Ga(CH_2SiMe_3)_3$ (0.17g, 0.5 mmol) in hexane (10 mL), a hexane solution of pyridazine (0.04 g, 0.5 mmol in 5 mL hexane) was added *via* syringe at room temperature. As soon as pyridazine was added, a yellow suspension was formed which evolved into orange and then red solution. After stirring for 15 min at room temperature, PMDETA was added (0.11 mL, 0.5 mmol) followed by the complete removal of volatiles *in vacuo*. The residue was placed in glovebox, 9 mg of ferrocene was added as an internal standard and the mixture was dissolved in 1 mL of d_8-THF and sealed in Young's tap NMR tube.

The integration versus ferrocene revealed 50% of compound **3**, 36% of the second regioisomer (C4-gallated pyridazine) and 10% excess of GaR_3 reagent.

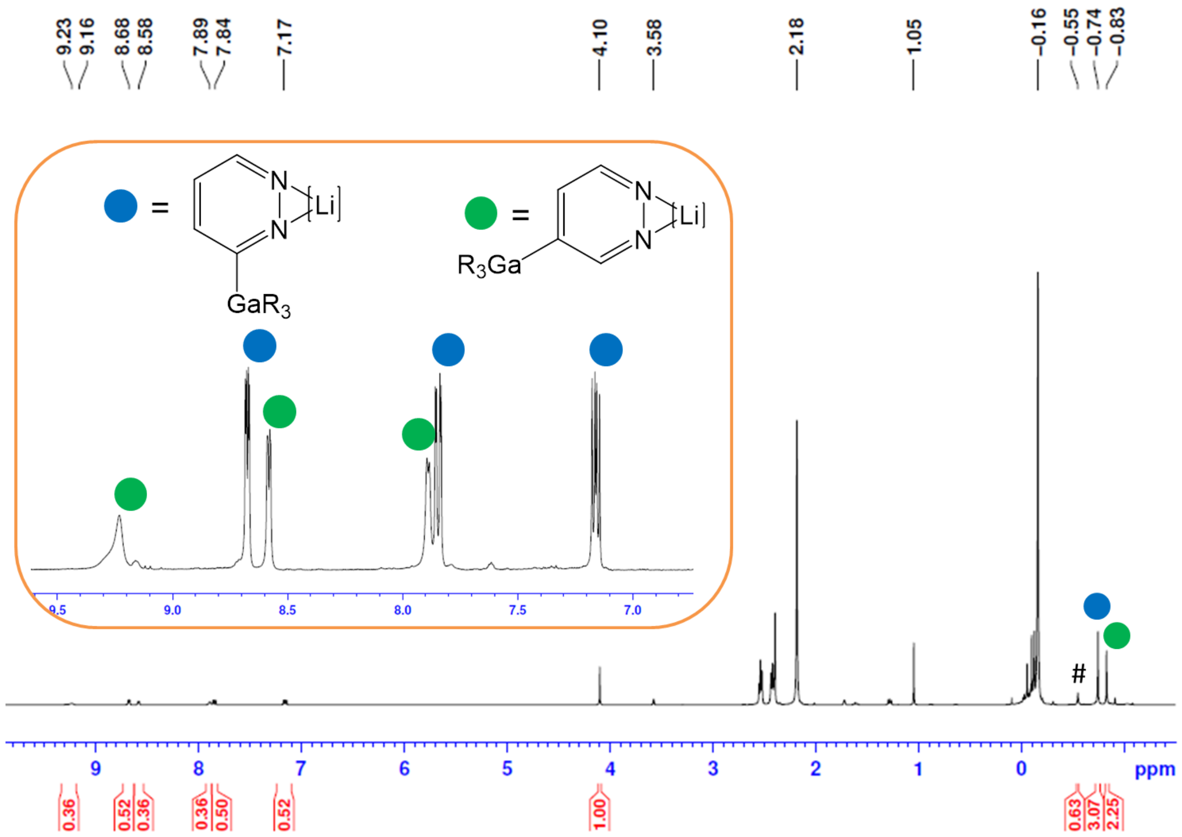

Figure S5: ^{1}H NMR of **3** (50 %) in d_8-THF solution.

4. Metallation of pyrimidine

a. The reaction was repeated exactly as described in preparation for compound **4** followed by the complete removal of volatiles *in vacuo*. The residue was placed in glovebox, 9 mg of ferrocene was added as an internal standard and the mixture was dissolved in 1 mL of d_8-THF and sealed in Young's tap NMR tube.

The integration *versus* ferrocene revealed 59% of compound **4**.

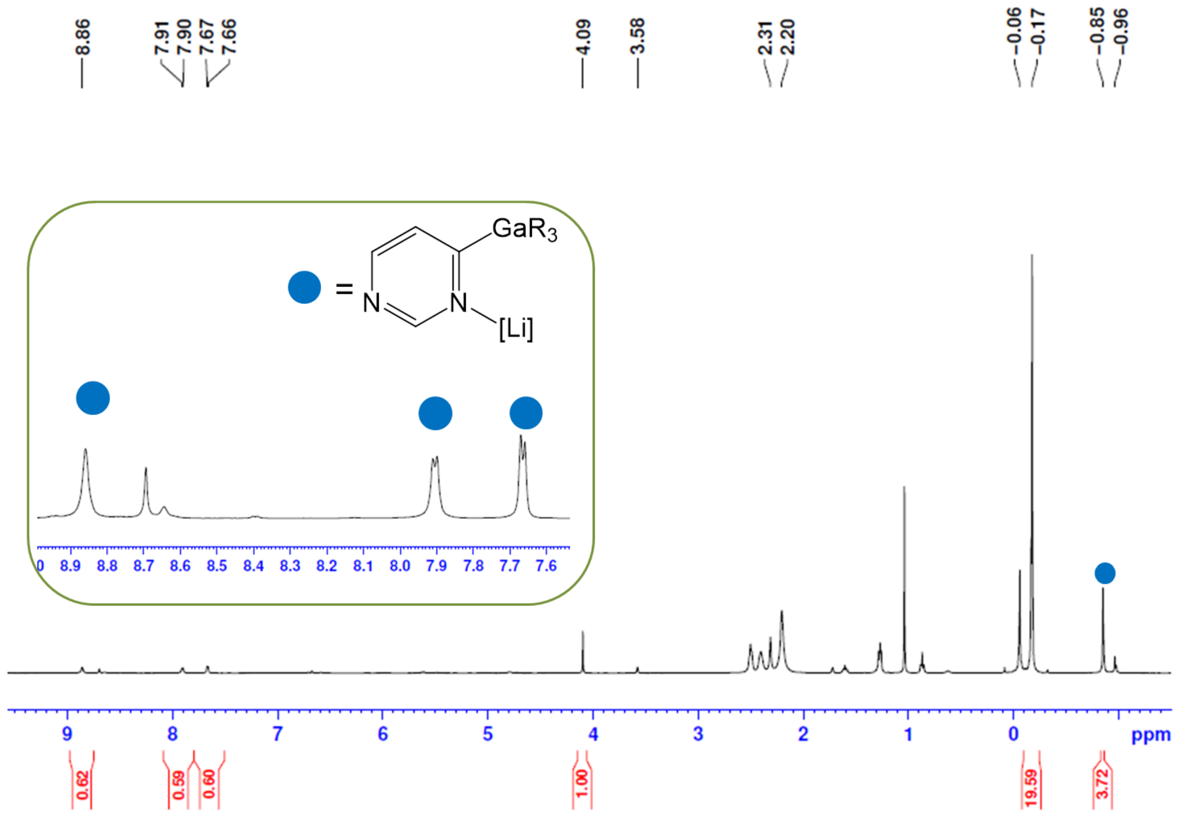

Figure S6: ^{1}H NMR of 4 (59%) in d_8-THF solution.

b. The reaction was performed with a variation in order of addition of reagents. To a hexane solution of pyrimidine (0.04 g, 0.5 mmol) and $Ga(CH_2SiMe_3)_3$ (0.17g, 0.5 mmol) in hexane (10 mL), LiTMP (0.07g, 0.5 mmol) was added *via* solid addition tube at room temperature. After stirring for 15 min at room temperature, PMDETA was added (0.11 mL, 0.5 mmol) followed by the complete removal of volatiles *in vacuo*. The residue was placed in glovebox, 9 mg of ferrocene was added as an internal standard and the mixture was dissolved in 0.7 mL of d_8-THF and sealed in Young's tap NMR tube.

The integration versus ferrocene revealed 43% of compound **4**.

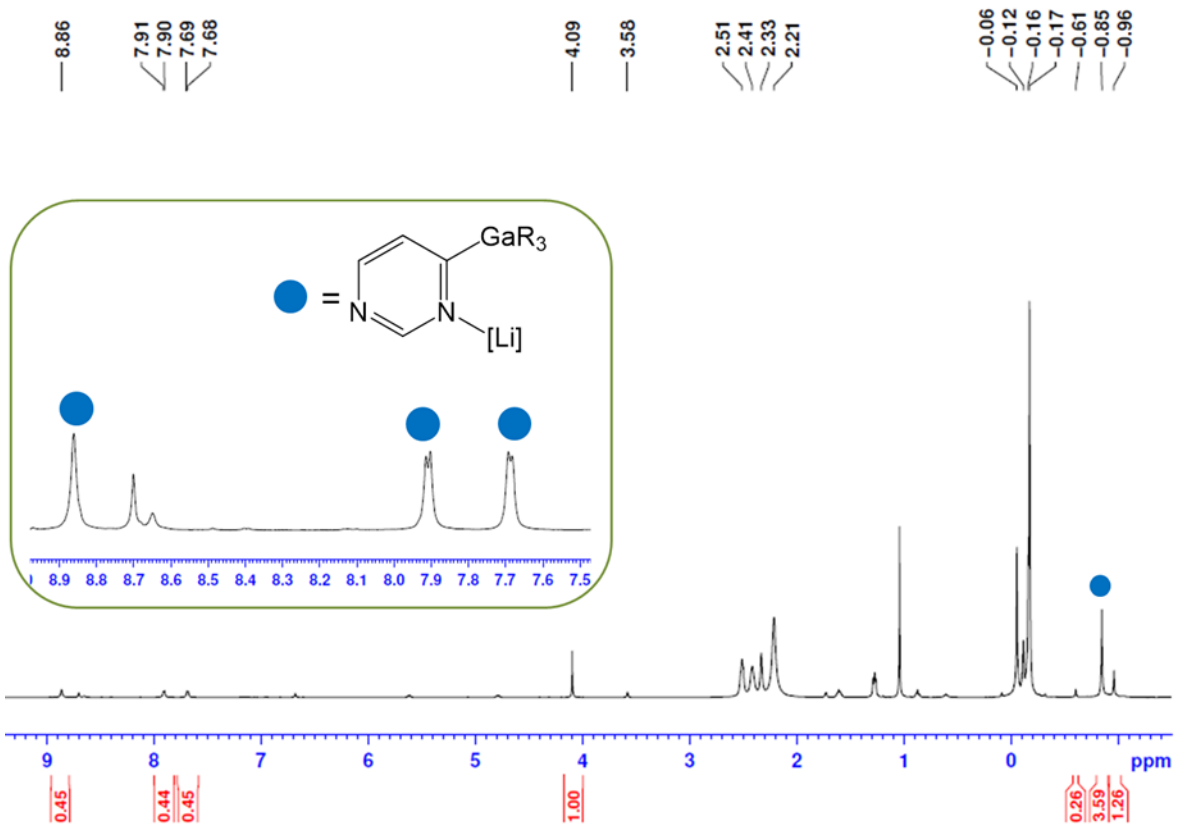

Figure S7: ^{1}H NMR of **4** (43%) in d_8-THF solution.

5. Metallation of benzothiazole

The reaction was repeated exactly as described in preparation for compound 5 followed by the complete removal of volatiles *in vacuo*. The residue was placed in glovebox, 9 mg of ferrocene was added as an internal standard and the mixture was dissolved in 1 mL of C_6D_6 and sealed in Young's tap NMR tube.

The integration *versus* ferrocene revealed quantitative formation of **5**.

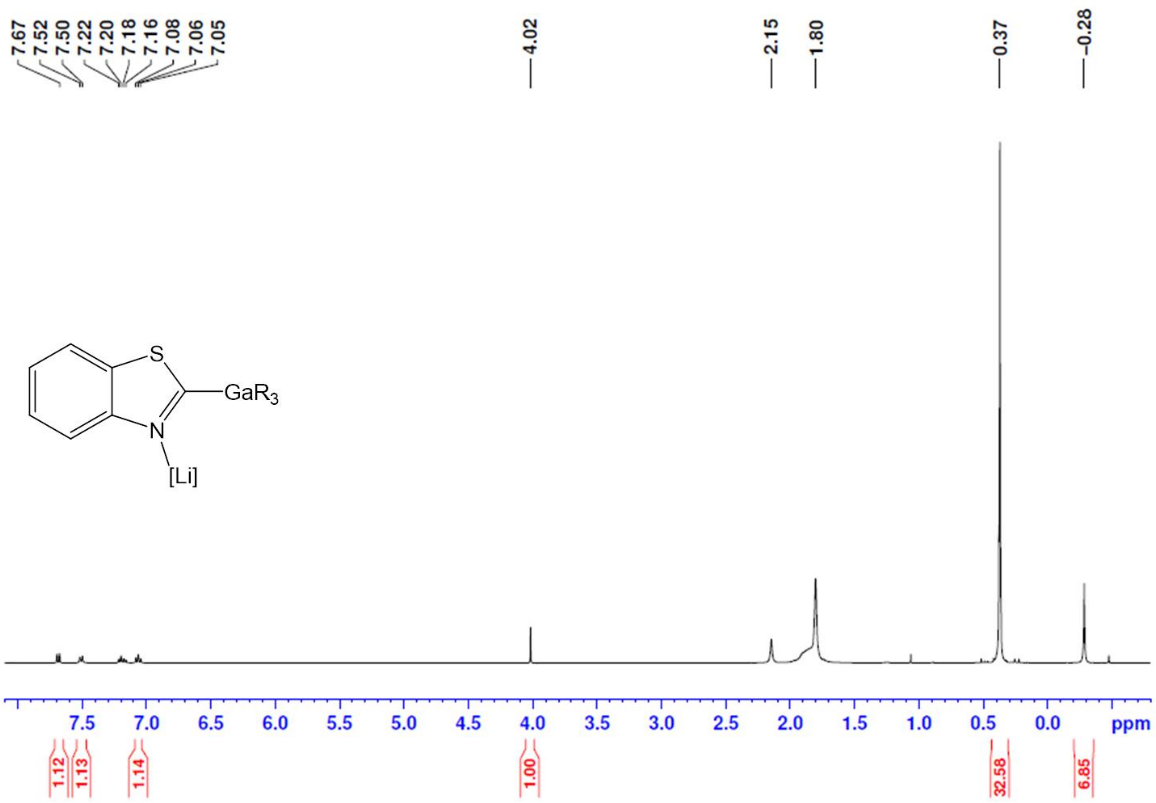

Figure S8: ^{1}H NMR of **5** (100%) in C_6D_6 solution.

6. $LiGaR_4$ with pyrazine

LiR (0.5 mmol, 0.5 mL of 1 M pentane solution) was added to a hexane solution of GaR_3 (0.5 mmol, 0.165 g in 10 mL hexane). A white, thick suspension was formed immediately and stirred for another hour. To this suspension, an equivalent of pyrazine (0.5 mmol, 0.04 g) was added via solid addition tube. The suspension turned yellow, then orange and was stirred for an hour. Finally, an equivalent of PMDETA (0.5 mmol, 0.11 mL) was added followed by a complete removal of volatiles *in vacuo*. The residue was placed in glovebox, 9 mg of ferrocene was added as an internal standard and the mixture was dissolved in 0.6 mL of d_8-THF and sealed in Young's tap NMR tube.

The integration *versus* ferrocene revealed 53% of compound incorporating dearomatized heterocycle.

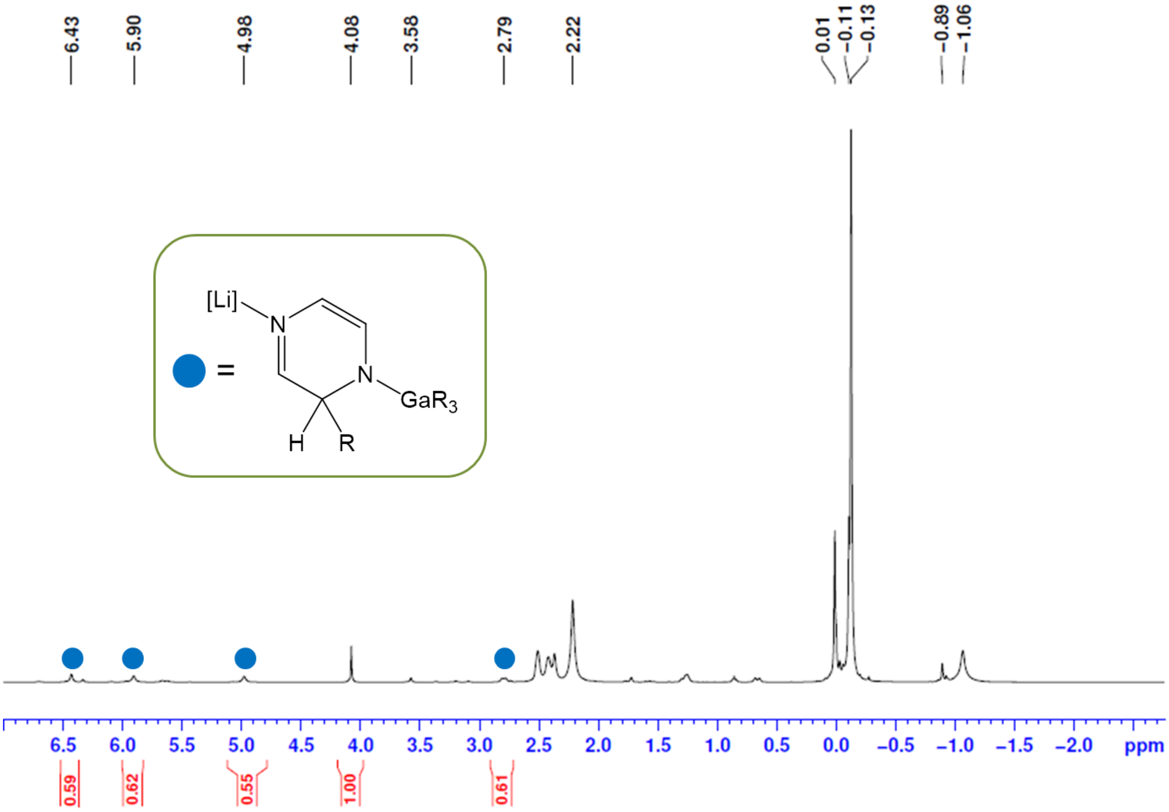

Figure S9: ^{1}H NMR of crude reaction mixture of $LiGaR_4$ and pyrazine in d_8-THF solution.

Solid state structures

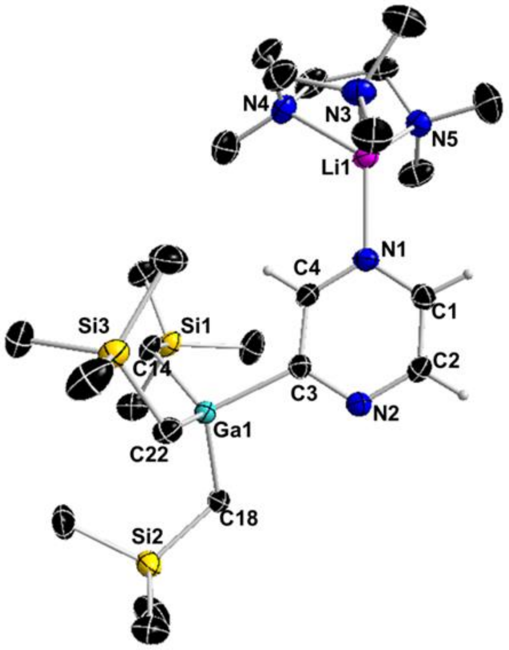

Figure S10: Molecular structure of **1** with 50% probability displacement ellipsoids. All hydrogen atoms except those on pyrazine have been omitted for clarity. Selected bond distances (Å) and bond angles (°): Ga(1)-C(3) 2.043(2), Ga(1)-C(14) 2.031(2), Ga(1)-C(18) 2.028(2), Ga(1)-C(22) 2.027(2), Li(1)-N(1) 2.020(4), Li(1)-N(3) 2.079(4), Li(1)-N(4) 2.098(4), Li(1)-N(5) 2.047(4), C(22)-Ga(1)-C(18) 110.40(9), C(22)-Ga(1)-C(14) 113.88(9), C(18)-Ga(1)-C(14) 112.94(9), C(22)-Ga(1)-C(3) 104.19(9), C(18)-Ga(1)-C(3) 108.29(9), C(14)-Ga(1)-C(3) 106.55(9).

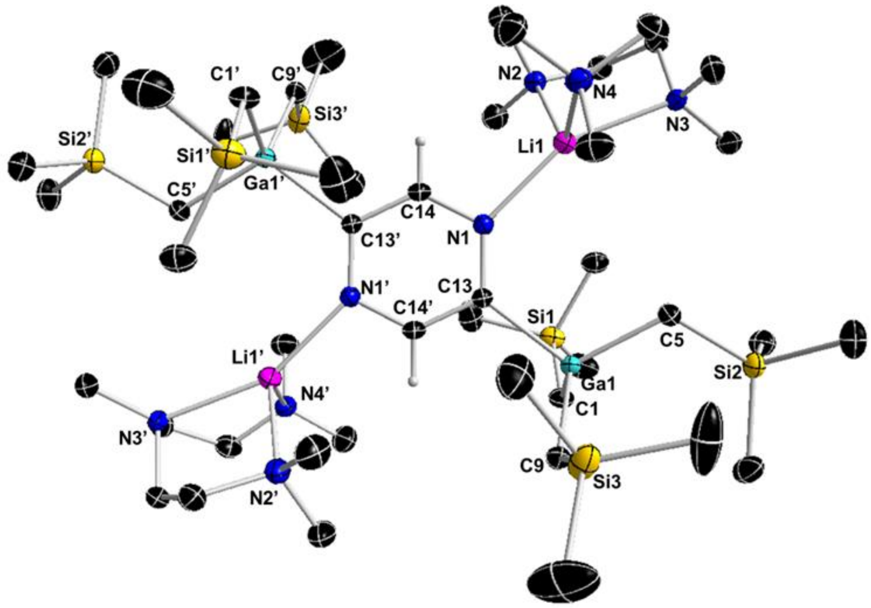

Figure S11: Molecular structure of **2** with 50% probability displacement ellipsoids. All hydrogen atoms except those on pyrazine ring have been omitted for clarity. Symmetry operator: -x, -y, -z. Selected bond distances (Å) and bond angles (°): Ga(1)-C(1) 2.022(3), Ga(1)-C(5) 2.021(2), Ga(1)-C(9) 2.018(3), Ga(1)-C(13) 2.062(3), Li(1)-N(1) 2.106(5), Li(1)-N(2) 2.197(5), Li(1)-N(3) 2.224(5), Li(1)-N(4) 2.144(5), C(5)-Ga(1)-C(13) 104.22(10), C(1)-Ga(1)-C(13) 100.99(10), C(9)-Ga(1)-C(5)

119.36(12), C(9)-Ga(1)-C(1) 114.86(10), C(5)-Ga(1)-C(1) 108.11(10), C(9)-Ga(1)-C(13) 107.12(12), N(1)-Li(1)-N(4) 108.2(2), N(1)-Li(1)-N(2) 103.7(2), N(4)-Li(1)-N(2) 126.8(2), N(1)-Li(1)-N(3) 154.5(3), N(4)-Li(1)-N(3) 84.22(18), N(2)-Li(1)-N(3) 84.74(17).

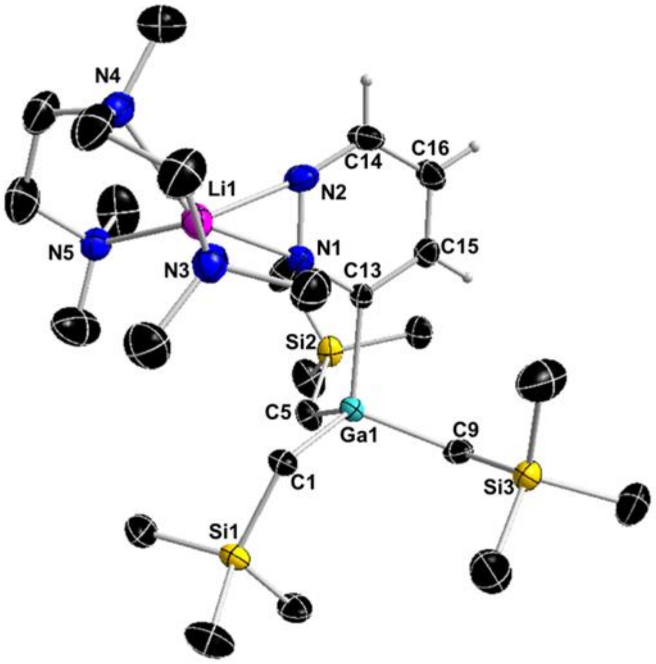

Figure S12: Molecular structure of **3** with 50% probability displacement ellipsoids. All hydrogen atoms except those on pyridazine ring have been omitted for clarity. The unit cell of **3** contains two crystallographically independent molecules with identical connectivity. One of these molecules contains minor disorder in the PMDETA ligand, thus structural discussion is focused on the non-disordered molecule. Selected bond distances (Å) and bond angles (°): Ga(1)-C(1) 2.018(2), Ga(1)-C(9) 2.023(2), Ga(1)-C(5) 2.031(2), Ga(1)-C(13) 2.056(2), Li(1)-N(1) 2.093(5), Li(1)-N(2) 2.043(5), Li(1)-N(3) 2.129(5), Li(1)-N(4) 2.169(5), Li(1)-N(5) 2.107(5), C(1)-Ga(1)-C(9) 113.96(9), C(1)-Ga(1)-C(5) 112.79(10), C(9)-Ga(1)-C(5) 107.71(10), C(1)-Ga(1)-C(13) 105.11(9), C(5)-Ga(1)-C(13) 110.16(9), C(9)-Ga(1)-C(13) 104.91(9), N(2)-Li(1)-N(1) 38.40(11), N(2)-Li(1)-N(5) 121.2(2), N(1)-Li(1)-N(5) 109.8(2), N(2)-Li(1)-N(3) 113.8(2), N(1)-Li(1)-N(3) 107.4(2), N(5)-Li(1)-N(3) 124.3(2), N(2)-Li(1)-N(4) 109.0(2), N(1)-Li(1)-N(4) 147.4(2), N(5)-Li(1)-N(4) 84.38(18), N(3)-Li(1)-N(4) 85.94(18).

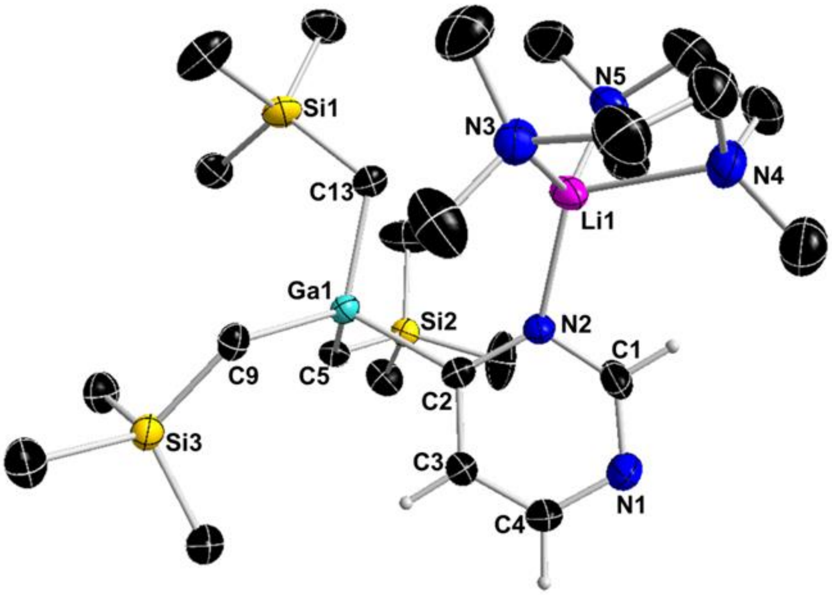

Figure S13: Molecular structure of **4** with 50% probability displacement ellipsoids. All hydrogen atoms except those on pyrimidine ring have been omitted for clarity. Selected bond distances (Å) and bond angles (°): Ga(1)-C(2) 2.052(32), Ga(1)-C(5) 2.031(3), Ga(1)-C(9) 2.030(3), Ga(1)-C(13) 2.020(3), Li(1)-N(2) 2.067(6), Li(1)-N(3) 2.157(7), Li(1)-N(4) 2.246(7), Li(1)-N(5) 2.162(7), C(5)-Ga(1)-C(2) 113.00(13), C(9)-Ga(1)-C(2) 108.09(13), C(13)-Ga(1)-C(2) 107.52(13), C(9)-Ga(1)-C(5) 113.97(13), C(13)-Ga(1)-C(5) 111.77(13), C(13)-Ga(1)-C(9) 111.84(14), N(2)-Li(1)-N(3) 111.8(3), N(2)-Li(1)-N(5) 123.0(3), N(3)-Li(1)-N(5) 123.9(3), N(2)-Li(1)-N(4) 113.9(3), N(3)-Li(1)-N(4) 85.0(2), N(5)-Li(1)-N(4) 83.5(2).

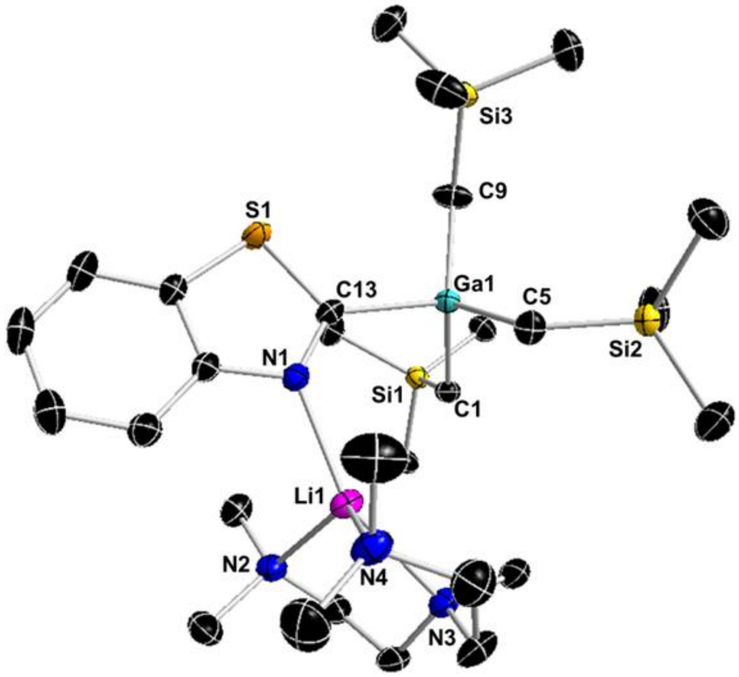

Figure S14: Molecular structure of **5** with 50% probability displacement ellipsoids. All hydrogen atoms have been omitted for clarity. Selected bond distances (Å) and bond angles (°): Ga(1)-C(1) 2.022(3), Ga(1)-C(5) 2.021(2), Ga(1)-C(9) 2.018(3), Ga(1)-C(13) 2.062(3), Li(1)-N(1) 2.016(5), Li(1)-N(2) 2.197(5), Li(1)-N(3) 2.224(5), Li(1)-N(4) 2.144(5), C(9)-Ga(1)-C(5) 119.36(12), C(9)-

Ga(1)-C(1) 114.86(10), C(5)-Ga(1)-C(1) 108.11(10), C(9)-Ga(1)-C(13) 107.12(12), C(5)-Ga(1)-C(13) 104.22(10), C(1)-Ga(1)-C(13) 100.99(10), N(1)-Li(1)-N(4) 108.2(2), N(1)-Li(1)-N(2) 103.7(2), N(4)-Li(1)-N(2) 126.8(2), N(1)-Li(1)-N(3) 154.5(3), N(4)-Li(1)-N(3) 84.22(18), N(2)-Li(1)-N(3) 84.74(17)

NMR spectra of products

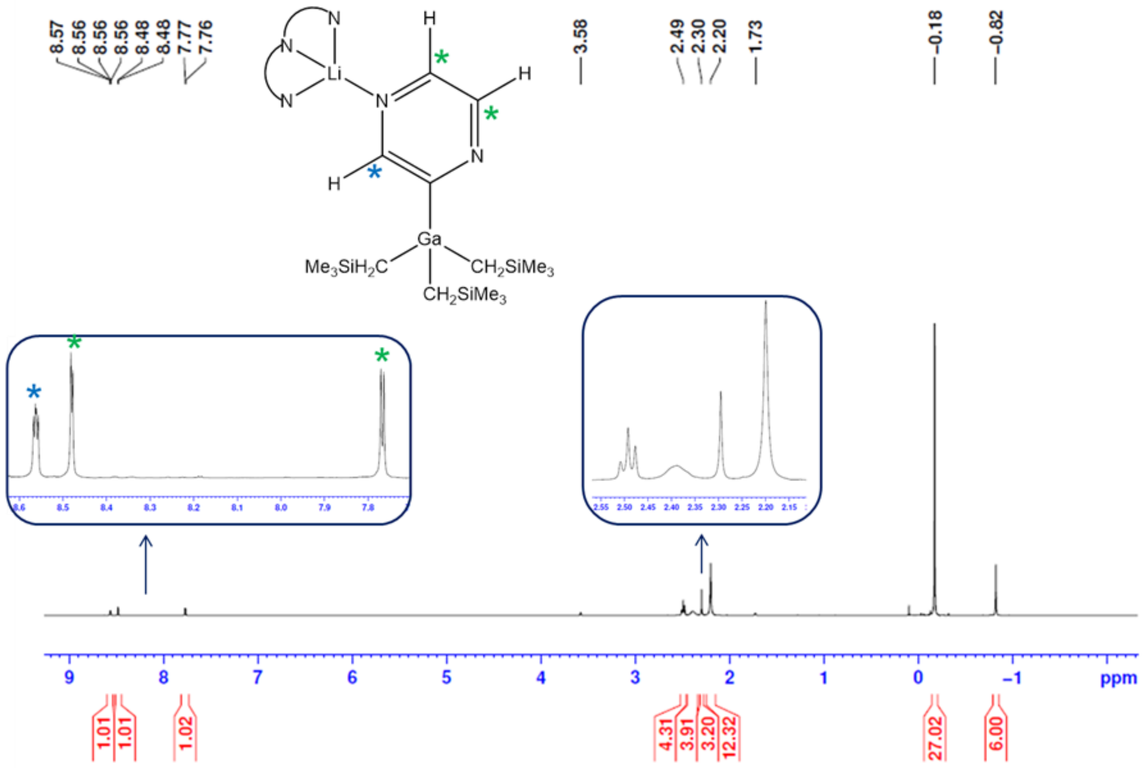

Figure S15: ^{1}H NMR spectrum of crystalline **1** in d_8-THF.

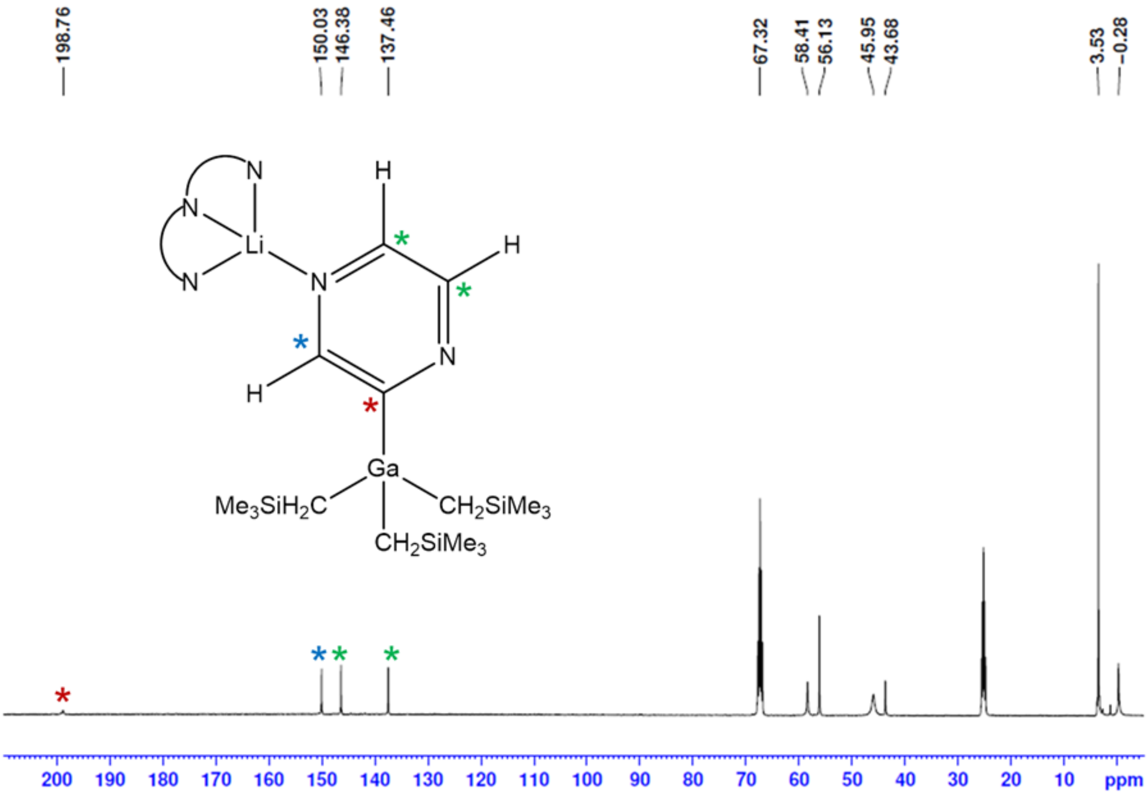

Figure S16: ^{13}C NMR spectrum of crystalline **1** in d_8-THF.

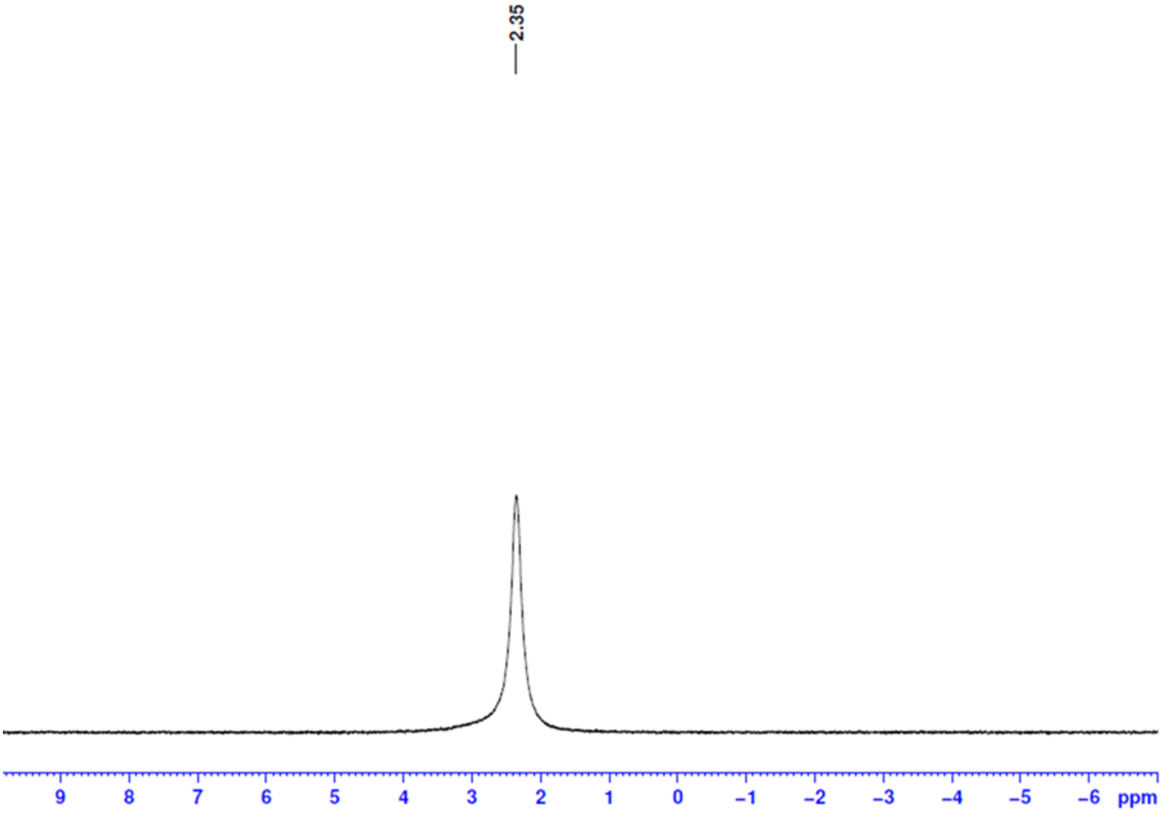

Figure S17: ^{7}Li NMR spectrum of crystalline **1** in d_8-THF.

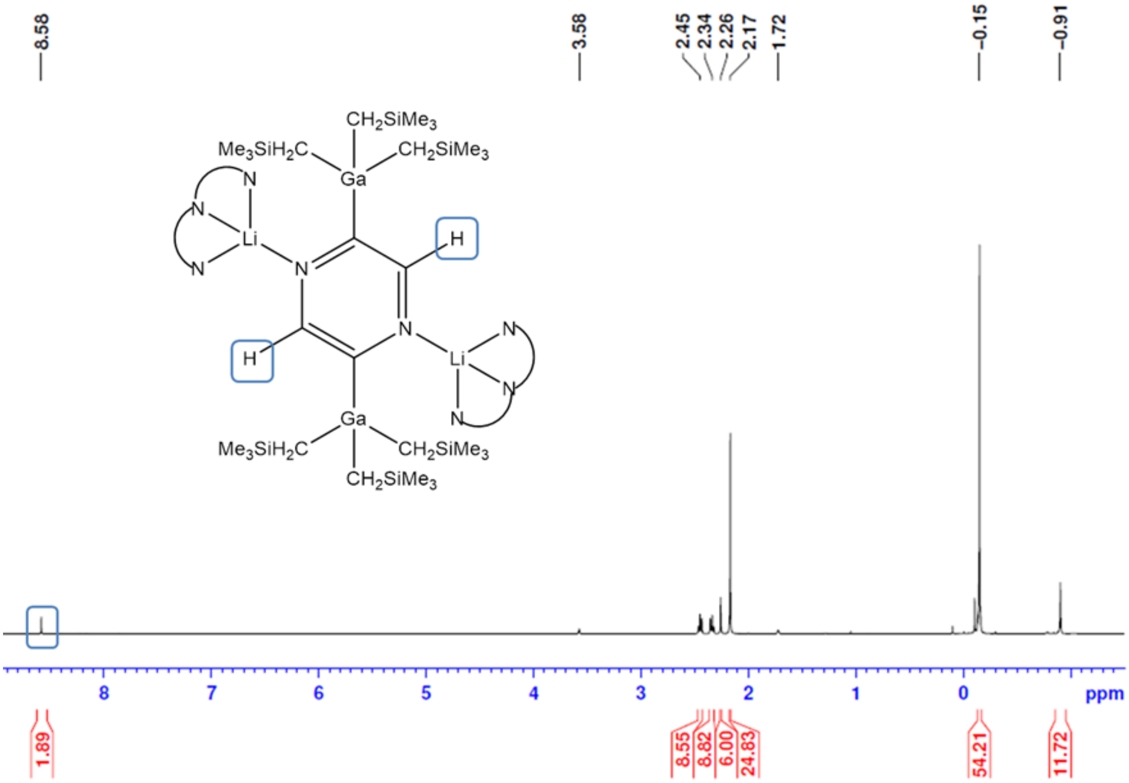

Figure S18: ^{1}H NMR spectrum of crystalline **2** in d_8-THF.

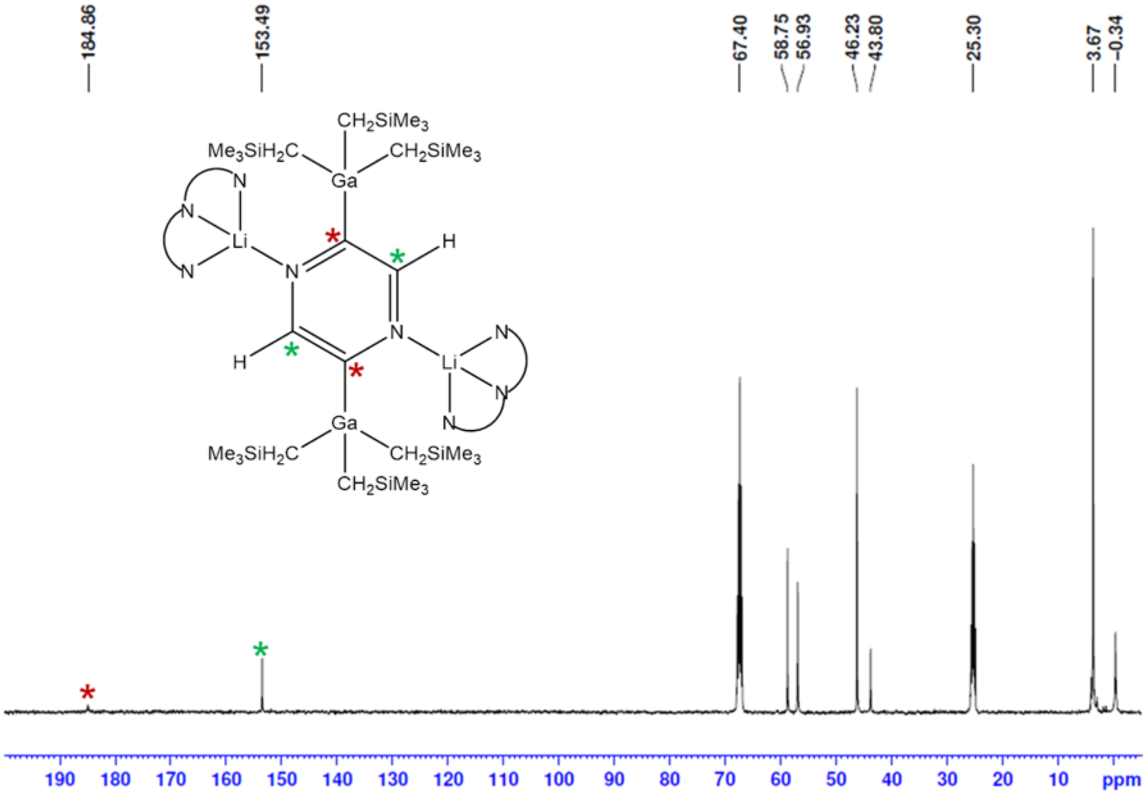

Figure S19: ^{13}C NMR spectrum of crystalline **2** in d_8-THF.

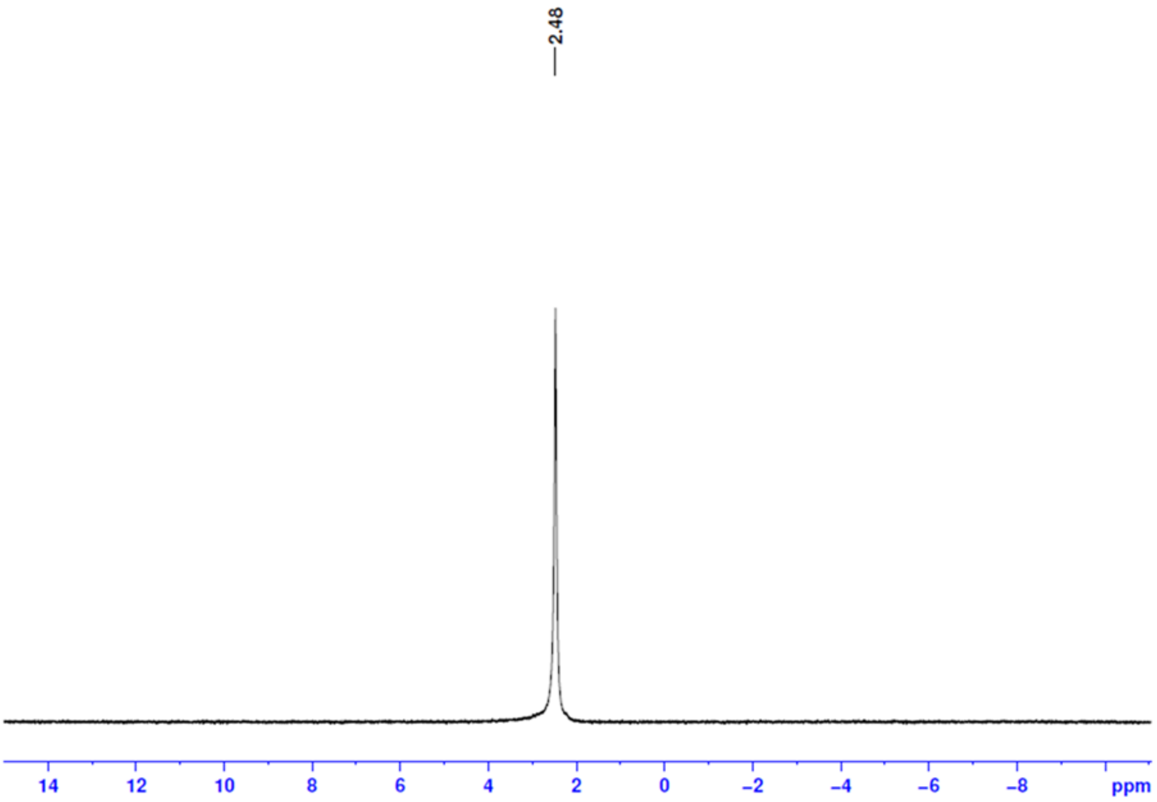

Figure S20: ^{7}Li NMR spectrum of crystalline **2** in d_8-THF.

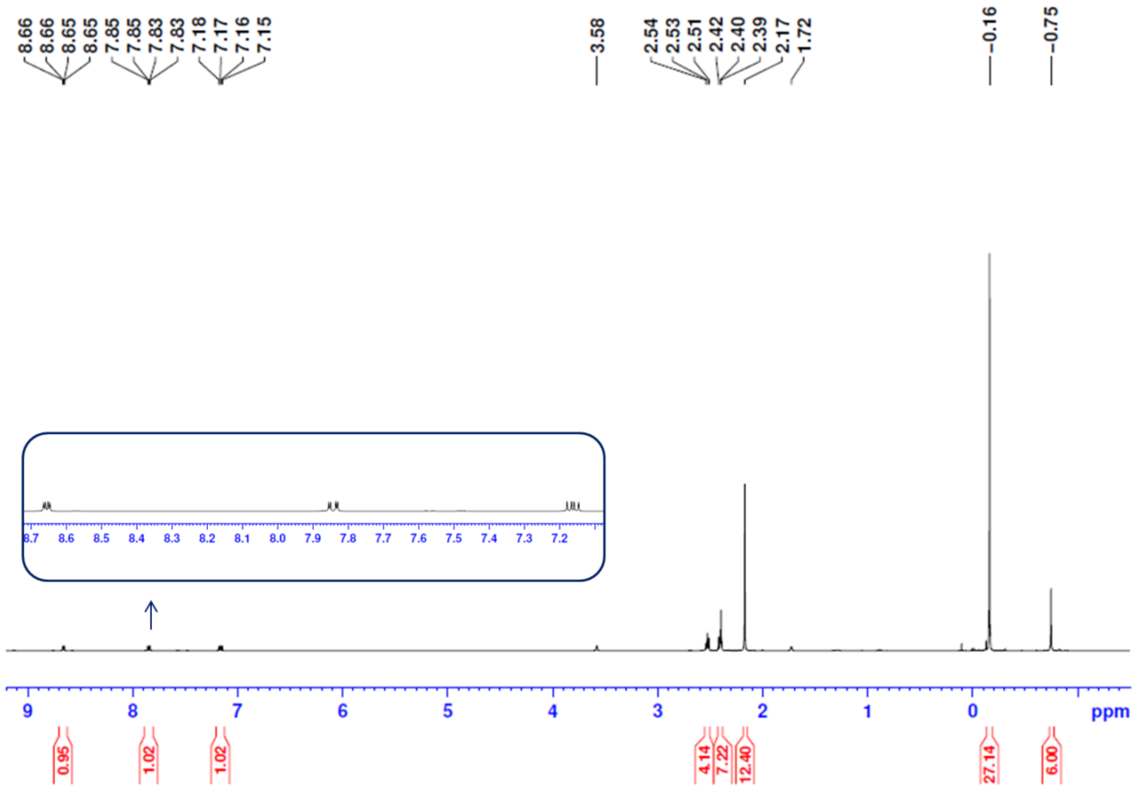

Figure S21: ^{1}H NMR of crystalline 3 in d_8-THF.

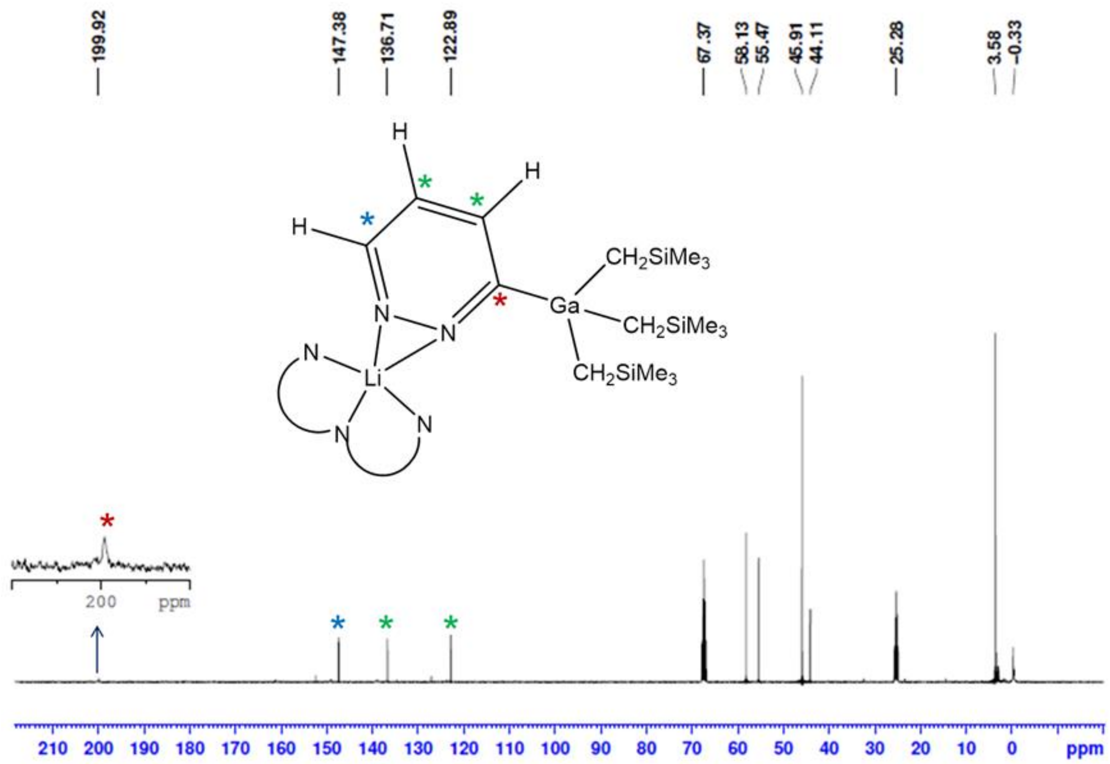

Figure S22: ^{13}C NMR spectrum of crystalline **3** in d_8-THF.

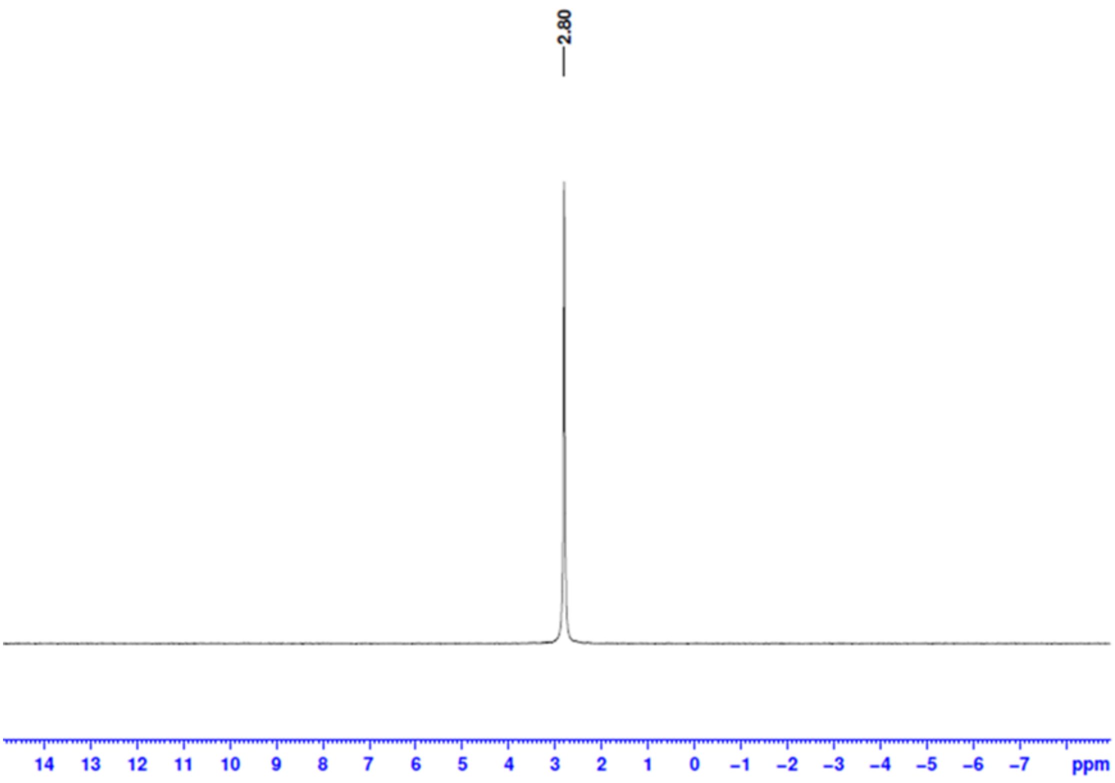

Figure S23: ^{7}Li NMR spectrum of crystalline **3** in d_8-THF.

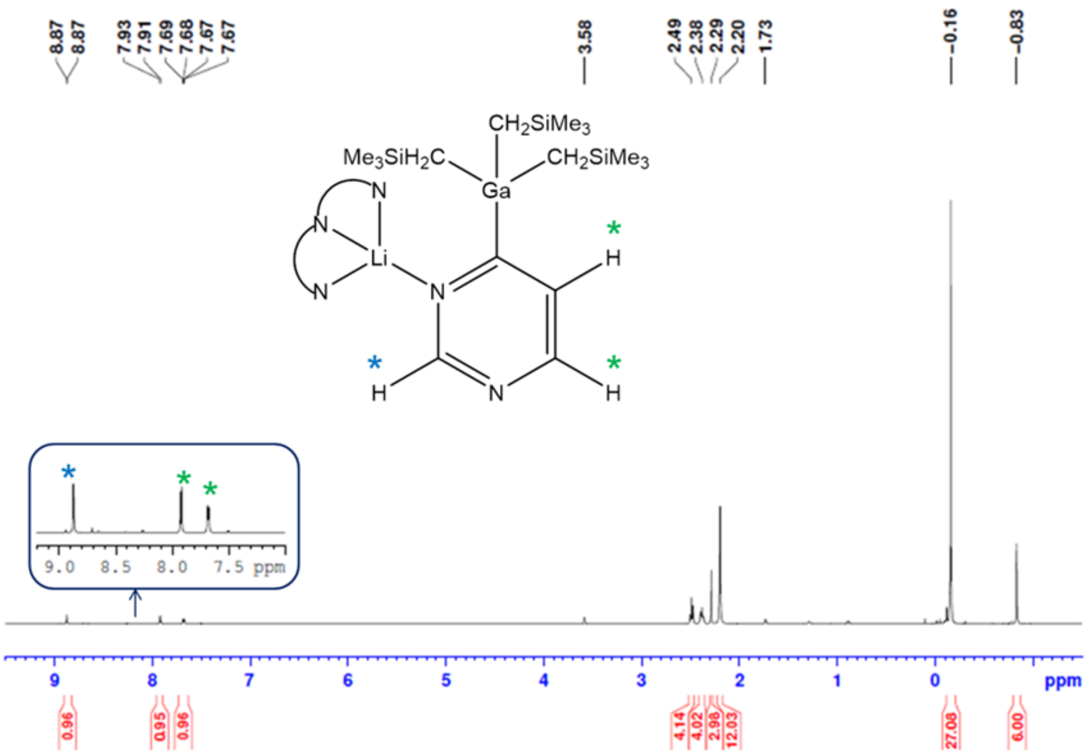

Figure S24: ^{1}H NMR spectrum of crystalline **4** in d_8-THF.

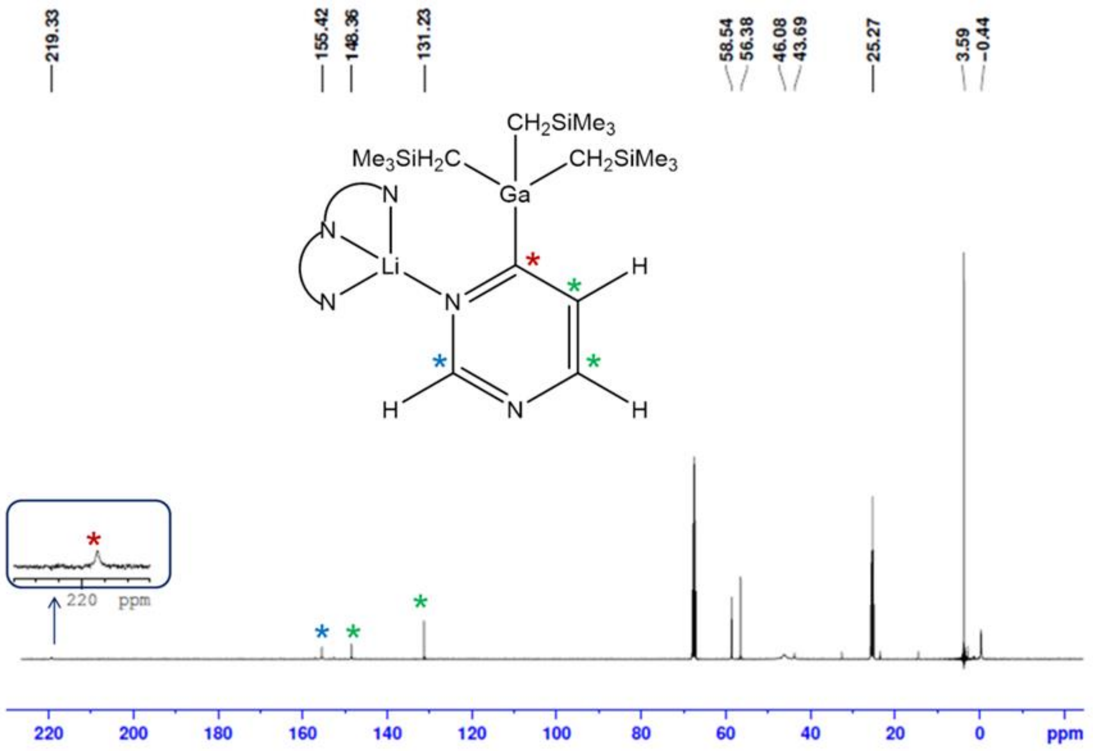

Figure S25: ^{13}C NMR spectrum of crystalline **4** in d_8-THF.

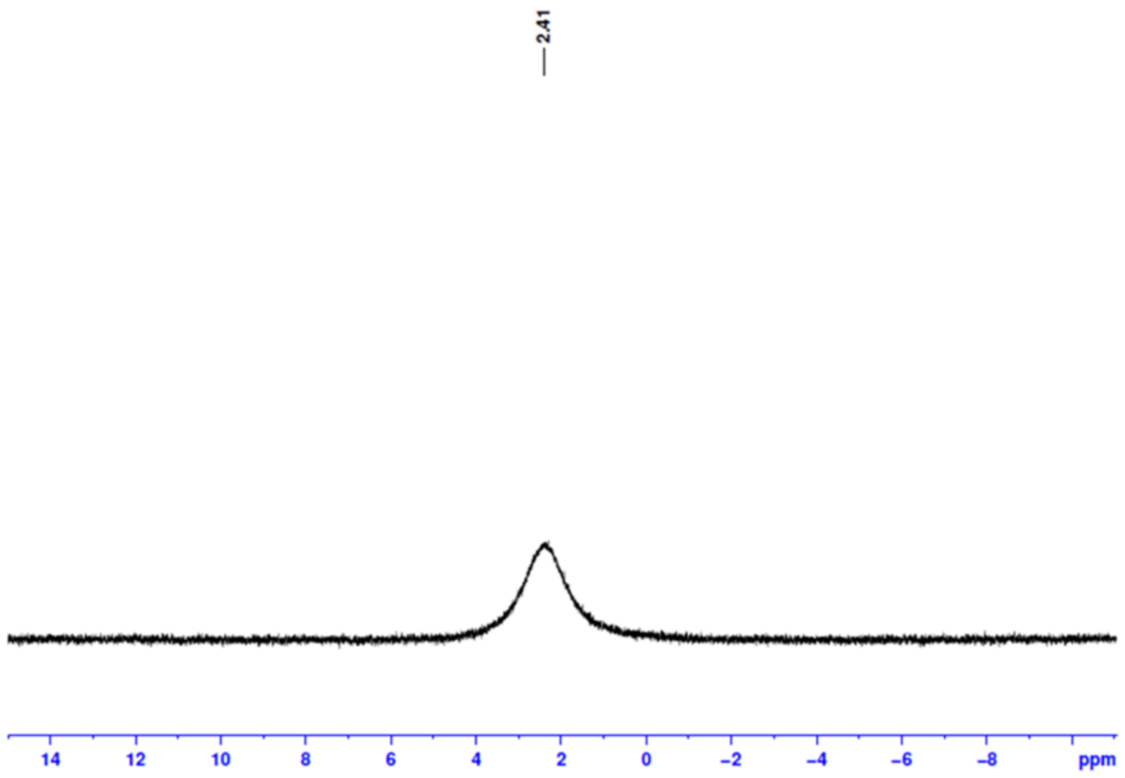

Figure S26: ^{7}Li NMR spectrum of crystalline **4** in d_8-THF.

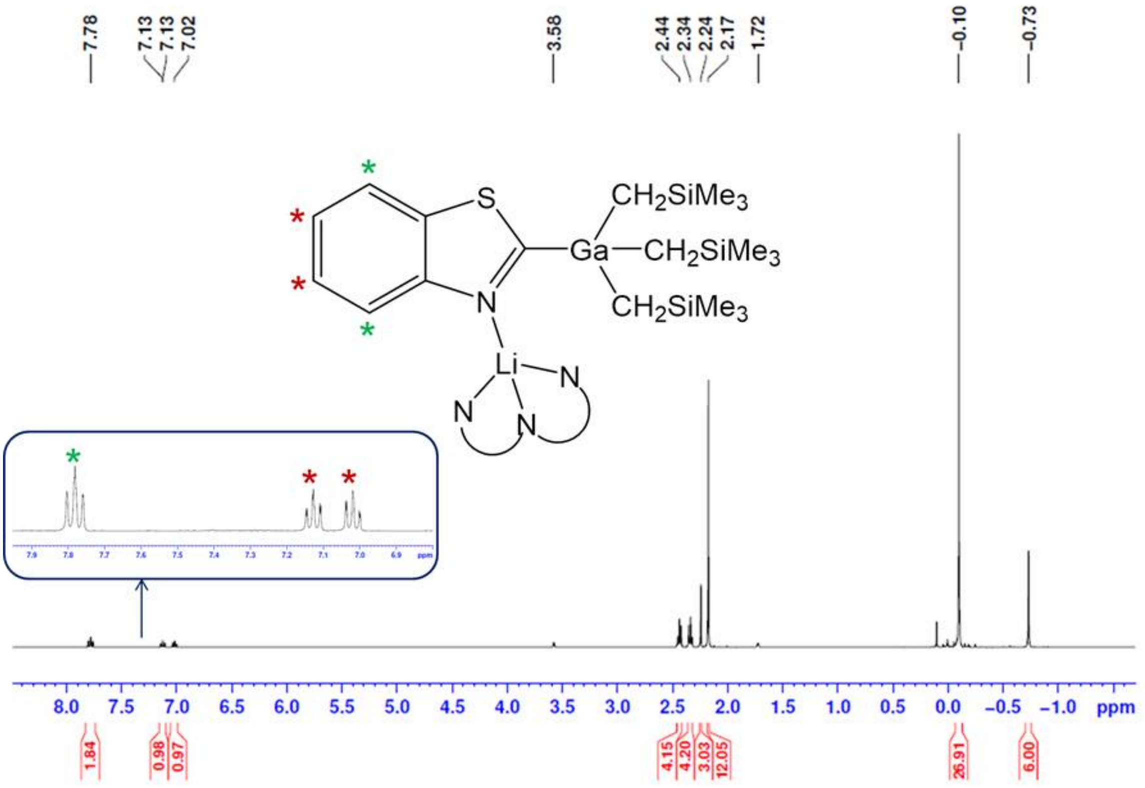

Figure S27: ^{1}H NMR spectrum of crystalline **5** in d_8-THF.

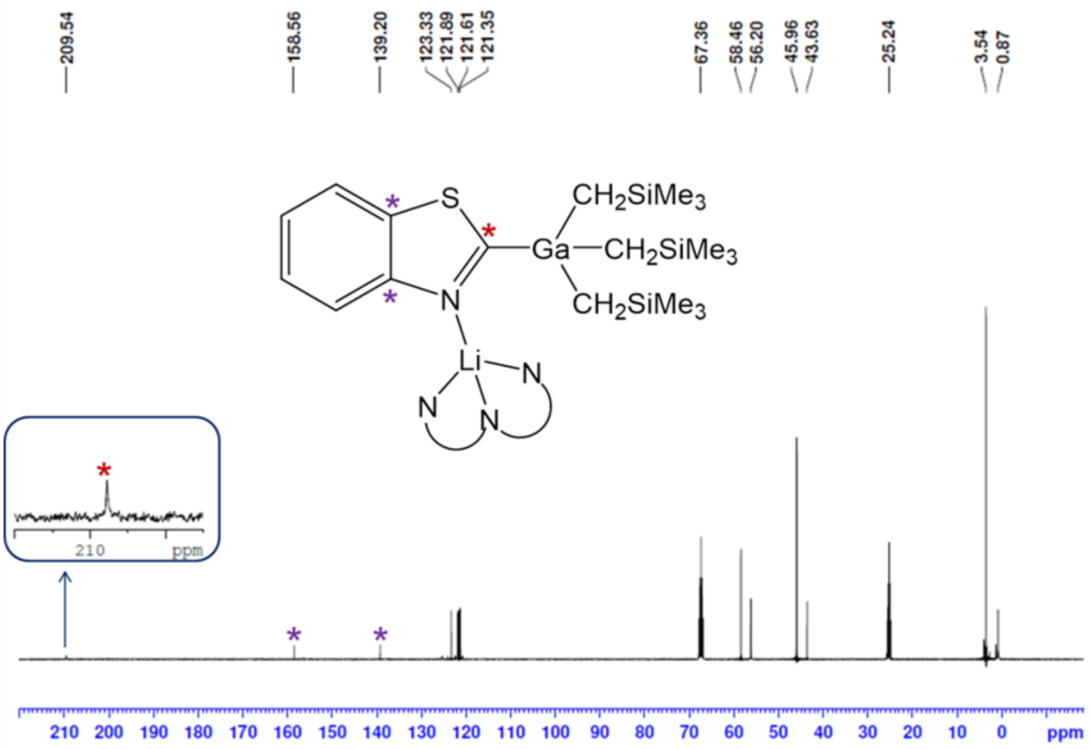

Figure S28: ^{13}C NMR spectrum of crystalline **5** in d_8-THF

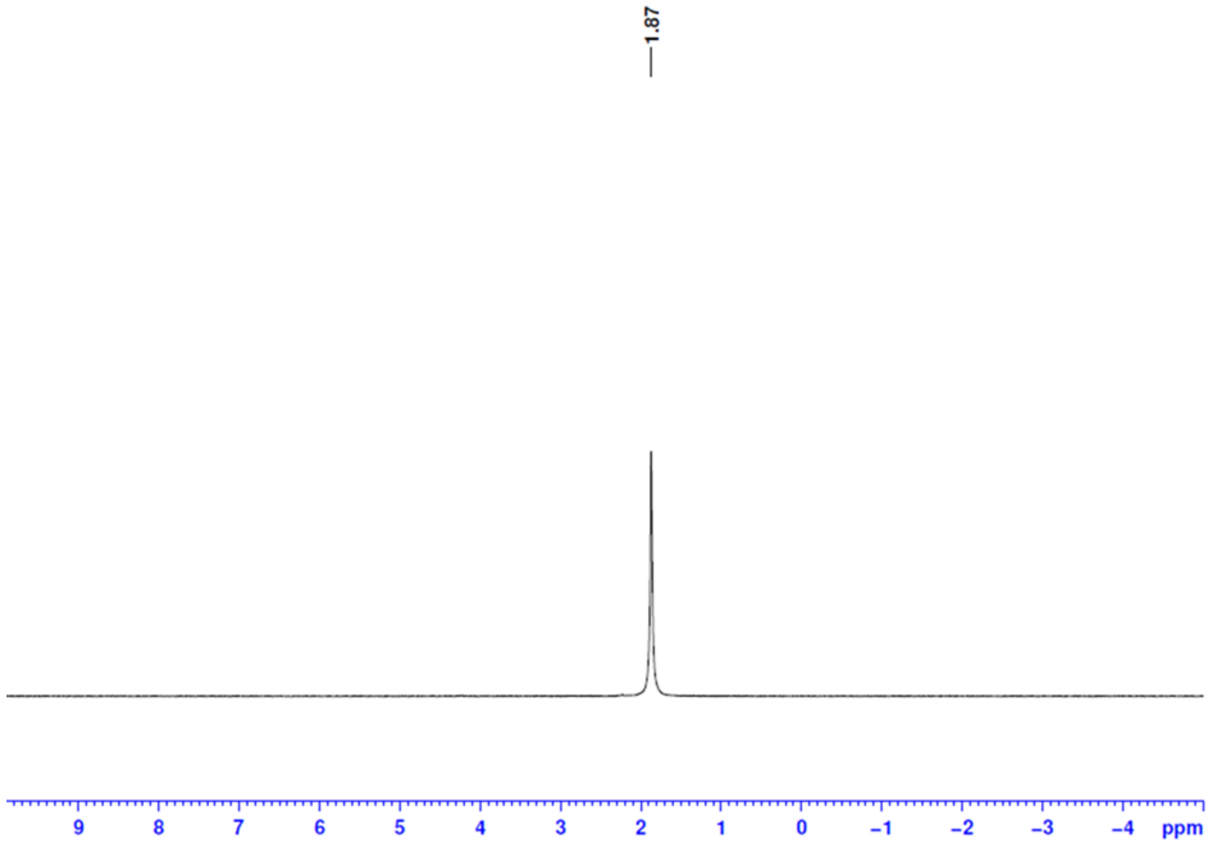

Figure S29: ^{7}Li NMR spectrum of crystalline **5** in d_8-THF.

Preliminary electrophilic quenching studies

1. Synthesis of **2-methylbenzothiazole** (**6**)

To a toluene solution (10 mL) of crystalline [2-(GaR$_3$)-3-{Li(PMDETA)}C$_6$H$_4$NCS] (**5**) (0.5 mmol, 0.322 g), MeOTf was added (4 mmol, 0.33 g) at -70 °C. The reaction mixture was stirred for 1 hour at -70 °C and another hour at room temperature. After the filtration, all volatiles were removed *in vacuo*. The residue was placed in glovebox, 10 mg of ferrocene was added as an internal standard and the mixture was dissolved in C$_6$D$_6$ and sealed in Young's tap NMR tube.

The integration *versus* ferrocene revealed 62% of compound **6** and 8% (hydrolysis).

^{1}H NMR (298 K, C$_6$D$_6$) δ(ppm) 3.54 (3H, s, C*H*$_3$), 6.74 (1H, d, CH-btz), 6.83 (1H, t, CH-btz), 6.91 (1H, t, CH-btz), 7.02 (1H, t, C*H*-btz), **^{13}C{^{1}H} NMR (298 K, C$_6$D$_6$)** 39.1 (*C*H$_3$), 114.1 (*C*H-btz), 122.7 (*C*H-btz), 125.7 (*C*H-btz), 127.1 (*C*H-btz), 134.2 (*C*-btz), 144.8 (*C*-btz), 158.1 (*C*-Ga).

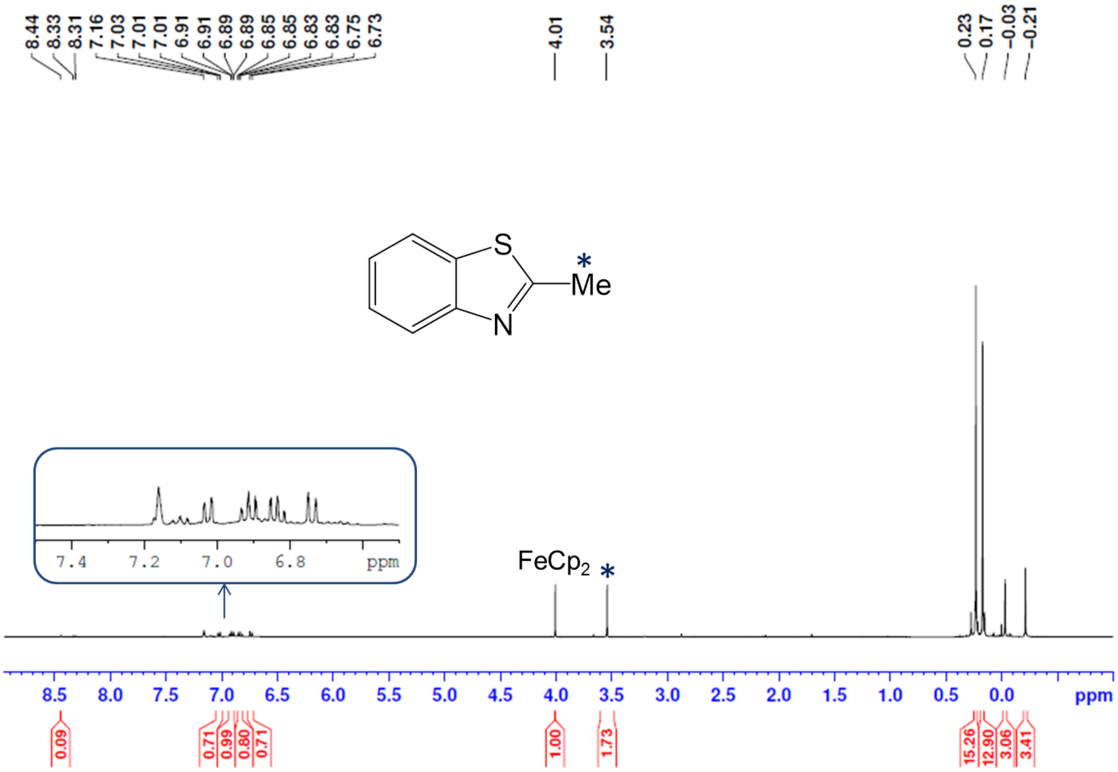

Figure S30: ^{1}H NMR of **6** (62%) in C_6D_6 solution.

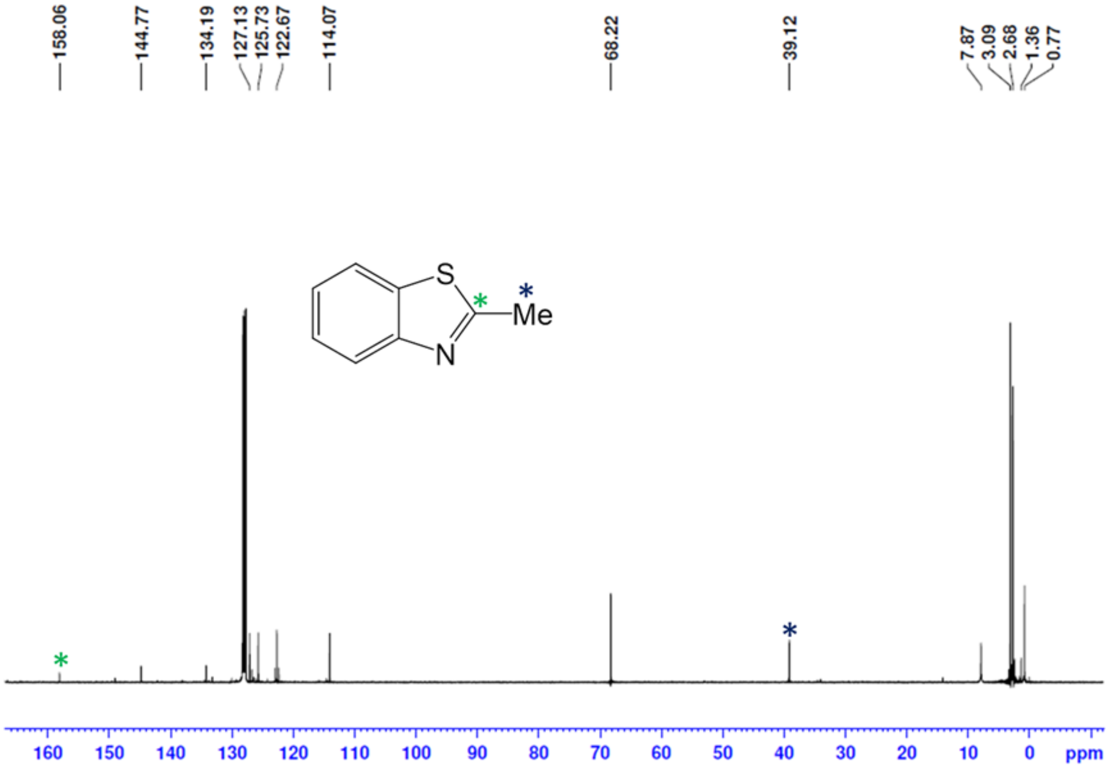

Figure 31: ^{13}C NMR spectrum of **6** in C_6D_6 solution.

2. Synthesis of **2-(trimethylsilyl)benzothiazole** (**7**)

To a toluene solution (10 mL) of crystalline [2-(GaR$_3$)-3-{Li(PMDETA)}C$_6$H$_4$NCS] (**5**) (0.5 mmol, 0.322 g) , Me$_3$SiCl was added (3 mmol, 0.38 mL). The reaction mixture was stirred for 2 hours at room temperature followed by the complete removal of volatiles *in vacuo*. The residue was placed in glovebox, 9 mg of ferrocene was added as an internal standard and the mixture was dissolved in C$_6$D$_6$ and sealed in Young's tap NMR tube.

The integration *versus* ferrocene revealed 88% of compound **7** and 12% (hydrolysis). Side-products (PMDETA and Me$_3$Si-CH$_2$SiMe$_3$) are also visible in the spectra.

^{1}H NMR (298 K, C$_6$D$_6$) δ(ppm) 0.34 (9H, s, SiC*H*$_3$), 7.10 (1H, t, C*H*-btz), 7.17 (1H, t, C*H*-btz), 7.65 (1H, d, C*H*-btz), 78.16(1H, d, C*H*-btz). **^{13}C{^{1}H} NMR (298 K, C$_6$D$_6$)** -1.2 (Si*C*H$_3$), 121.9 (*C*H-btz), 123.8 (*C*H-btz), 125.2 (*C*H-btz), 125.9 (*C*H-btz), 136.7 (*C*-btz), 157.0 (*C*-btz), 176.0 (*C*-Ga).

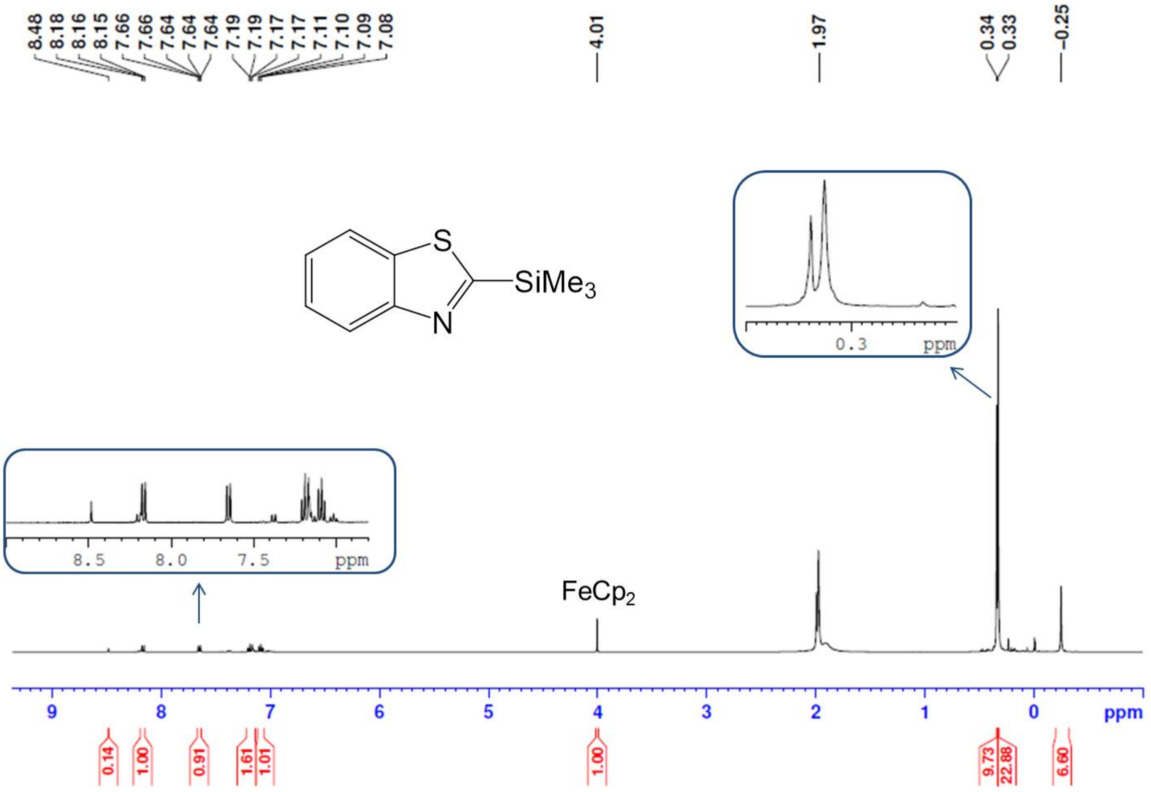

Figure S32: ^{1}H NMR spectrum of **7** (88%) in C$_6$D$_6$ solution.

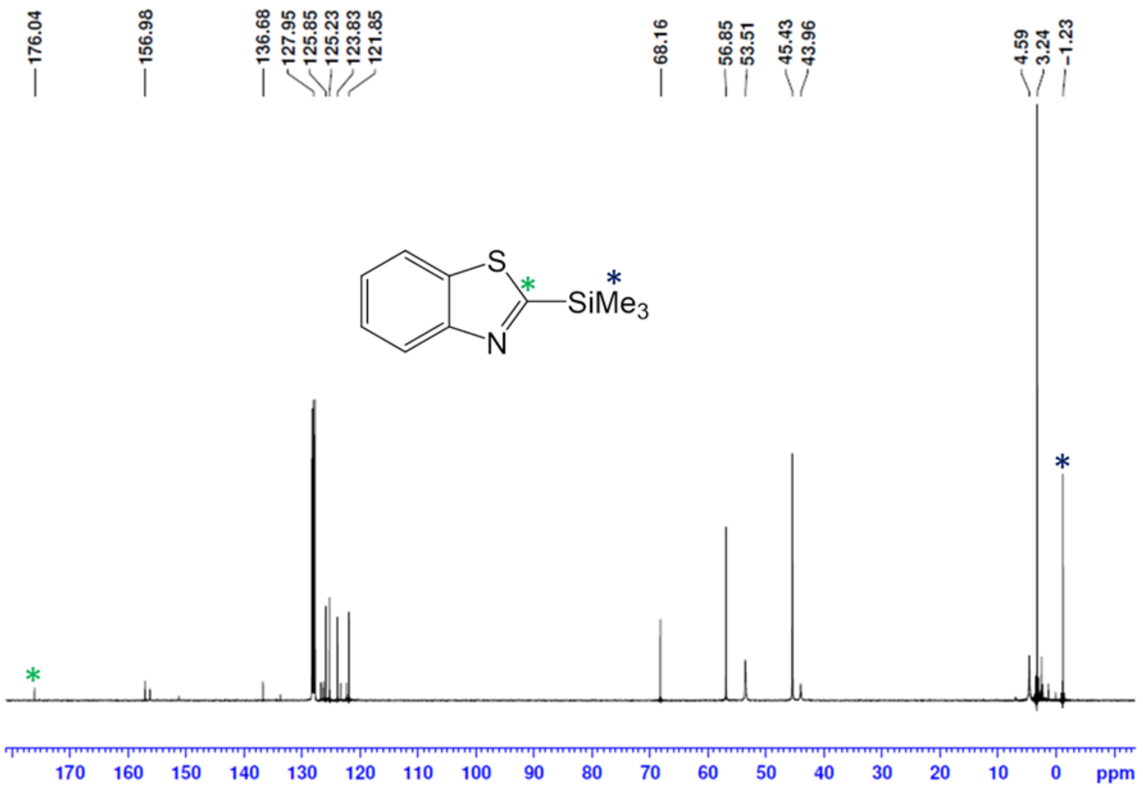

Figure S33: ^{13}C NMR spectrum of **7** in C_6D_6 solution.

3. Synthesis of **2-(trimethylsilyl)pyrazine** (**8**)

To a toluene solution (10 mL) of crystalline [1-(PMDETA)Li-3-(GaR$_3$)-$C_4H_3N_2$] (**1**) (0.5 mmol, 0.295 g) , Me_3SiCl was added (3 mmol, 0.38 mL). The reaction mixture was stirred for 2 hours at room temperature followed by the complete removal of volatiles *in vacuo*. The residue was placed in glovebox, 9 mg of ferrocene was added as an internal standard and the mixture was dissolved in C_6D_6 and sealed in Young's tap NMR tube.

The integration *versus* ferrocene revealed 59% of compound **8**. Side-products (PMDETA and Me_3Si-CH_2-$SiMe_3$) are also visible in the spectra.

^{1}H NMR (298 K, C_6D_6) δ(ppm) -0.82 (9H, s, C*H*$_3$Si), 7.94 (1H, s, pyrazine), 8.28 (1H, mult, pyrazine), 8.59 (1H, s, pyrazine). **^{13}C{^{1}H} NMR (298 K, C_6D_6)** -2.3 (*C*H$_3$Si), 141.1 (*C*H-pyrazine), 146.0 (*C*H-pyrazine), 147.3 (*C*H-pyrazine), 165.9 (*C*-SiMe$_3$).

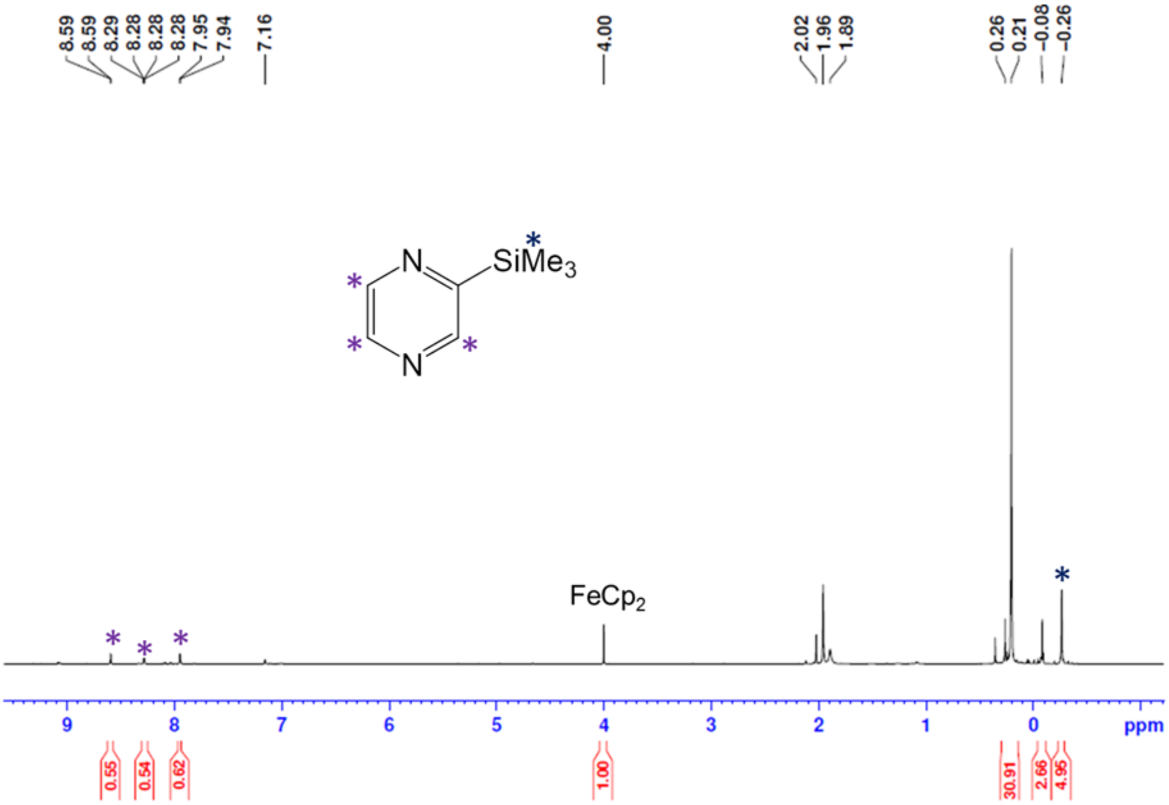

Figure S34: ^{1}H NMR spectrum of **8** (59%) in C_6D_6 solution.

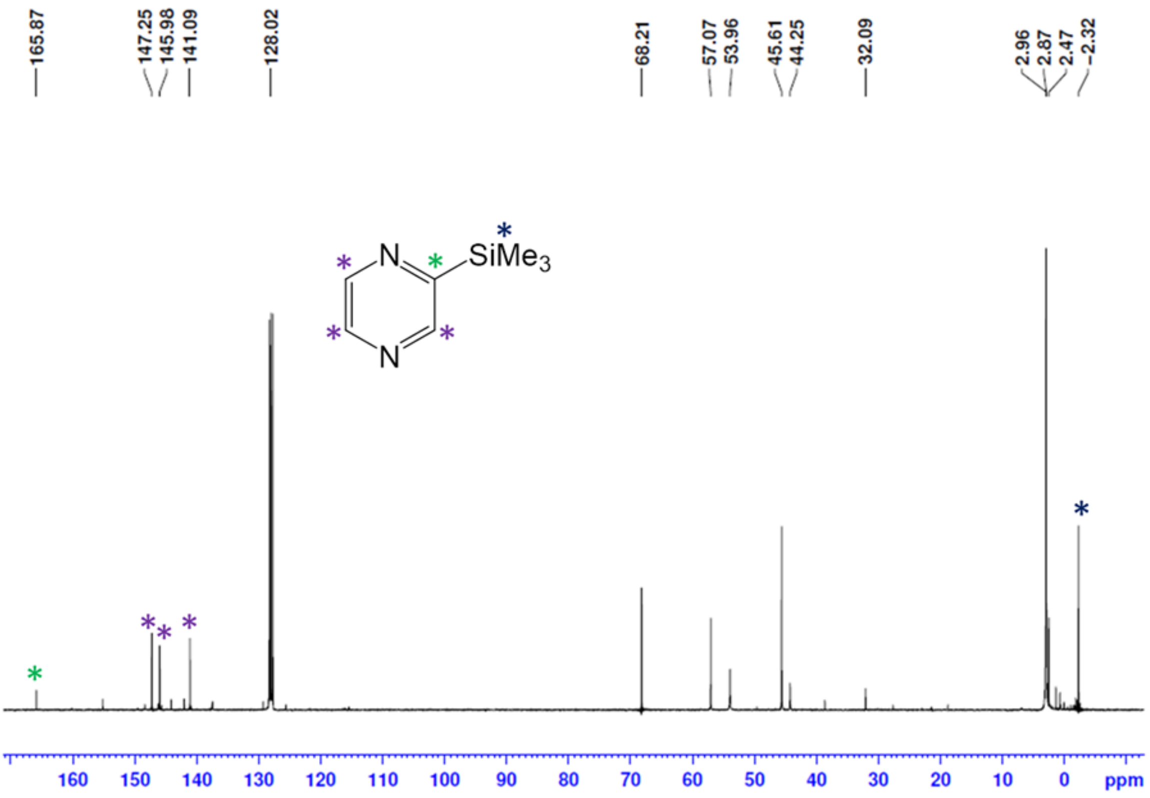

Figure S35: ^{13}C NMR spectrum of **8** in C_6D_6 solution.
